# The heterogenous and diverse population of prophages in *Mycobacterium* genomes

**DOI:** 10.1128/msystems.00446-23

**Published:** 2023-10-04

**Authors:** Lawrence Abad, Christian H. Gauthier, Isabella Florian, Deborah Jacobs-Sera, Graham F. Hatfull

**Affiliations:** 1 Department of Biological Sciences, University of Pittsburgh, Pittsburgh, Pennsylvania, USA; Los Alamos National Laboratory, Los Alamos, New Mexico, USA

**Keywords:** bacteriophages, prophages, *Mycobacterium*

## Abstract

Bacterial genomes commonly carry one or more integrated prophages, although prophage prevalence varies greatly in different bacterial genera and species. We have used the recently developed DEPhT software to discover prophages present in sequenced *Mycobacterium* genomes. Prophages are unevenly distributed among mycobacteria; *Mycobacterium tuberculosis* strains are devoid of intact prophages, whereas 75% of *Mycobacterium abscessus-chelonae* strains carry at least one prophage, and some have up to nine. Newly discovered prophage sequences are generally distinct from those of lytic phages isolated using *Mycobacterium smegmatis* as a host, although assembly of over 3,800 phage and prophage genomes into clusters based on shared sequence similarity yields seven clusters containing both phage and prophage genomes. *M. abscessus* prophages use a usually large repertoire of *attB* sites for prophage integration, and new integration-proficient vectors demonstrate the functionality of integration systems identified *in silico* and provide tools useful for advancing *M. abscessus* genetics. Genomic analysis reveals additional accessory and likely mobile parts of *Mycobacterium* genomes including defective prophages, prophage-like regions, and satellite elements proposed to be candidate phage-inducible chromosomal islands (PICIs). *M. abscessus* prophages and PICIs are unusual in that they often encode phage-encoded ESX-secreted toxin (PEST) systems that are located adjacent to the phage integration functions and are lysogenically expressed and likely provide a fitness advantage to their host.

**IMPORTANCE:**

*Mycobacterium* species include several human pathogens and mycobacteriophages show potential for therapeutic use to control *Mycobacterium* infections. However, phage infection profiles vary greatly among *Mycobacterium abscessus* clinical isolates and phage therapies must be personalized for individual patients. *Mycobacterium* phage susceptibility is likely determined primarily by accessory parts of bacterial genomes, and we have identified the prophage and phage-related genomic regions across sequenced *Mycobacterium* strains. The prophages are numerous and diverse, especially in *M. abscessus* genomes, and provide a potentially rich reservoir of new viruses that can be propagated lytically and used to expand the repertoire of therapeutically useful phages.

## INTRODUCTION

Temperate bacteriophages are common, and their prevalence is reflected in the abundance of prophages in sequenced bacterial genomes (1, 2). Most of these prophages are integrated site-specifically into the host chromosome, although some replicate as plasmids extrachromosomally (3, 4). Prophages can influence the physiology of their bacterial hosts including altered metabolism and pathogenicity, but their prevalence varies substantially among different bacterial species, and often between different strains of a bacterial species (5). They also confer defense against phages closely related to themselves (homotypic defense) and can often defend against infection by genetically unrelated phages (heterotypic defense) (6, 7).

The phylum Actinobacteria includes many genera of environmental or clinical importance, one of the most prominent being species in the genus *Mycobacterium* (8). *Mycobacterium tuberculosis* is the causative agent of human tuberculosis (TB), a major human pathogen especially in the developing world, and widespread drug resistance is a major impediment to TB control (9). The nontuberculous mycobacteria (NTM) include opportunistic pathogens such as *Mycobacterium avium* and *Mycobacterium abscessus* which are often intrinsically resistant to antibiotics and are challenging to manage clinically, especially in people with cystic fibrosis (10). Tens of thousands of *M. tuberculosis* clinical isolates have been sequenced and shown to have relatively low genomic diversity (11). In contrast, NTM strains, especially *M. abscessus* (12) and *M. avium* (13) have considerable genomic diversity and variation in drug susceptibilities.

Mycobacteriophages—phages infecting *Mycobacterium* hosts—have been extensively characterized, and over 2,200 have been sequenced and analyzed by comparative genomics, although nearly all of these were isolated on *Mycobacterium smegmatis* (14). These phages span considerable genetic diversity, but the diversity is heterogenous and phages with closely related genomes can be sorted into groups referred to as clusters (Clusters A, B, C, etc.) based on a threshold of 35% shared gene content (15). There is substantial genomic variation within the clusters, and many can be subdivided into subclusters (Subclusters A1, A2, A3, etc.) according to their overall relationships (16). To date, there are a total of 31 clusters and seven “singletons,” phages with no known close relatives. Some of these phages appear to have relatively broad host ranges and also efficiently infect the slow-growing *M. tuberculosis*, and these lie within specific clusters or subclusters such as AB, A2, A3, G, K, and L2 (17, 18). Some of these also infect some strains of *M. abscessus*, although there is great variation in phage susceptibilities among *M. abscessus* clinical isolates (19).

Mycobacteriophages show considerable potential as therapeutic agents for treating NTM infections, especially those caused by *M. abscessus* (20, 21). Because of the variation in *M. abscessus* clinical isolates, any potential therapeutic use requires personalized matching of phages to bacterial strains (22, 23). Nonetheless, 20 patients have received phage therapies for such infections with favorable outcomes in a majority of cases (22, 24 – 27). The genetic basis of the specificity of phage infection of *M. abscessus* strains presents a major impediment to broadening the therapeutic use of phages (19) and the development of phage cocktails that would be active against a large majority of clinical isolates.

Characterization of 82 *M*. *abscessus* clinical isolates showed that 75% carry one or more prophages integrated into their chromosomes (6, 19), similar to other studies (28). The diversity of these prophages is considerable, matching or exceeding that of the lytically growing *M. smegmatis* phages, and are grouped similarly into clusters (MabA, MabB, etc.) and subclusters (MabA1, MabA2, etc.) (29). None of the prophages are closely related to *M. smegmatis* phages, although a subset meets the shared gene content threshold for inclusion in the same clusters (15). These prophages are of particular interest for two reasons: they may encode phage defense systems that directly determine phage infection profiles (6), and because of their therapeutic potential when recovered as lytically propagated phages (19, 29).

We recently described a bioinformatics approach to prophage discovery that exploits genomic signatures to distinguish between phage and bacterial genomes (30). Here, we identify over 2,500 prophages present in *Mycobacteriaceae* genomes, and integrate their genomic diversity with previously described *Mycobacterium* phages. Prophages are especially prevalent in *M. abscessus-chelonae* clade strains, and these are replete with phage-encoded ESX-secreted toxin (PEST) systems. The expanded repertoire of *Mycobacterium* phage and prophage genomes facilitates discovery of an expanded collection of chromosomal satellite regions with features of prophage-induced chromosomal islands (PICIs), many of which also encode PEST systems.

## RESULTS

### Identification of NTM prophages

Using DEPhT (30), we searched for prophages in a total of 9,231 genomes in the family *Mycobacteriaceae* (31), most of which are *Mycobacterium* species but also include a small number of closely related *Hoyosella* strains (Table 1); in general, we used the taxonomic system set by Gupta et al. (31) but maintained the original species names (8, 32). This search identified 2,630 prophages. As the reliability of DEPhT to identify prophages suffers with low-quality assemblies (30), we limited our search to genome assemblies containing 100 contigs or fewer ( Data Set S1) and developed an additional method which supplements DEPhT and increases the accuracy of discovery in poorer assemblies. The Software for Phage-fragment Location In Contig Ends (SPLICE) in combination with DEPhT gives the most complete *Mycobacterium* prophage identification strategy to date, allowing for the discovery of an additional 278 prophages (~11%). The prophages identified using DEPhT are typically full length and intact, but those identified with DEPhT + SPLICE typically span two unjoined contigs, and thus are incomplete. Nonetheless, there is usually sufficient sequence information for preliminary cluster assignation. Each prophage is given a designation in the format: prophi*PATRICTaxon.PATRICStrain*-Prophage#. For example, two prophages present in *M. abscessus* (PATRIC taxon of 1761), strain number 28, would be designated prophi1761.28-1 and prophi1761.28-2. Prophages are listed in Data set S2.

**TABLE 1:** Average number of prophages discovered by DEPhT in clades of *Mycobacterium* and related bacterial species

Clade^*a*^	# genomes^*b*^	# genomes ≥1 prophage^*c*^	# prophages^*d*^	Mean # prophages^*e*^
*M. abscessus-chelonae* complex (*abscessus-chelonae*)	1,951	1,468	2,787	1.428
Other rapid-growing *Mycobacterium* (*fortuitum-vaccae*)	222	49	60	0.27
*M. terrae* complex (*terrae*)	18	2	3	0.167
*Hoyosella*	8	1	1	0.125
Slow-growing *Mycobacterium* (*tuberculosis-simiae*)	7,030	52	57	0.008
*M. triviale* group (*triviale*)	2	0	0	0
Totals^*f*^	9,231	1,572	2,908	0.315

^*a*^ Clades in the genus *Mycobacterium* and the closely related genus *Hoyosella*. Clades are as defined by Gupta et al. (31), and the clade names used by Gupta et al. are shown in parentheses.

^*b*^ The total numbers of genomes analyzed with DEPhT and SPLICE for each clade.

^*c*^ The total numbers of genomes with one or more prophages identified using DEPhT and SPLICE for each clade.

^*d*^ The total numbers of intact prophages identified using DEPhT and SPLICE for each clade of bacterium.

^*e*^ The average numbers of prophages for each clade are shown.

^*f*^ The total numbers of genomes and prophages identified from strains of *Mycobacterium* and *Hoyosella.*

**Fig 1:**
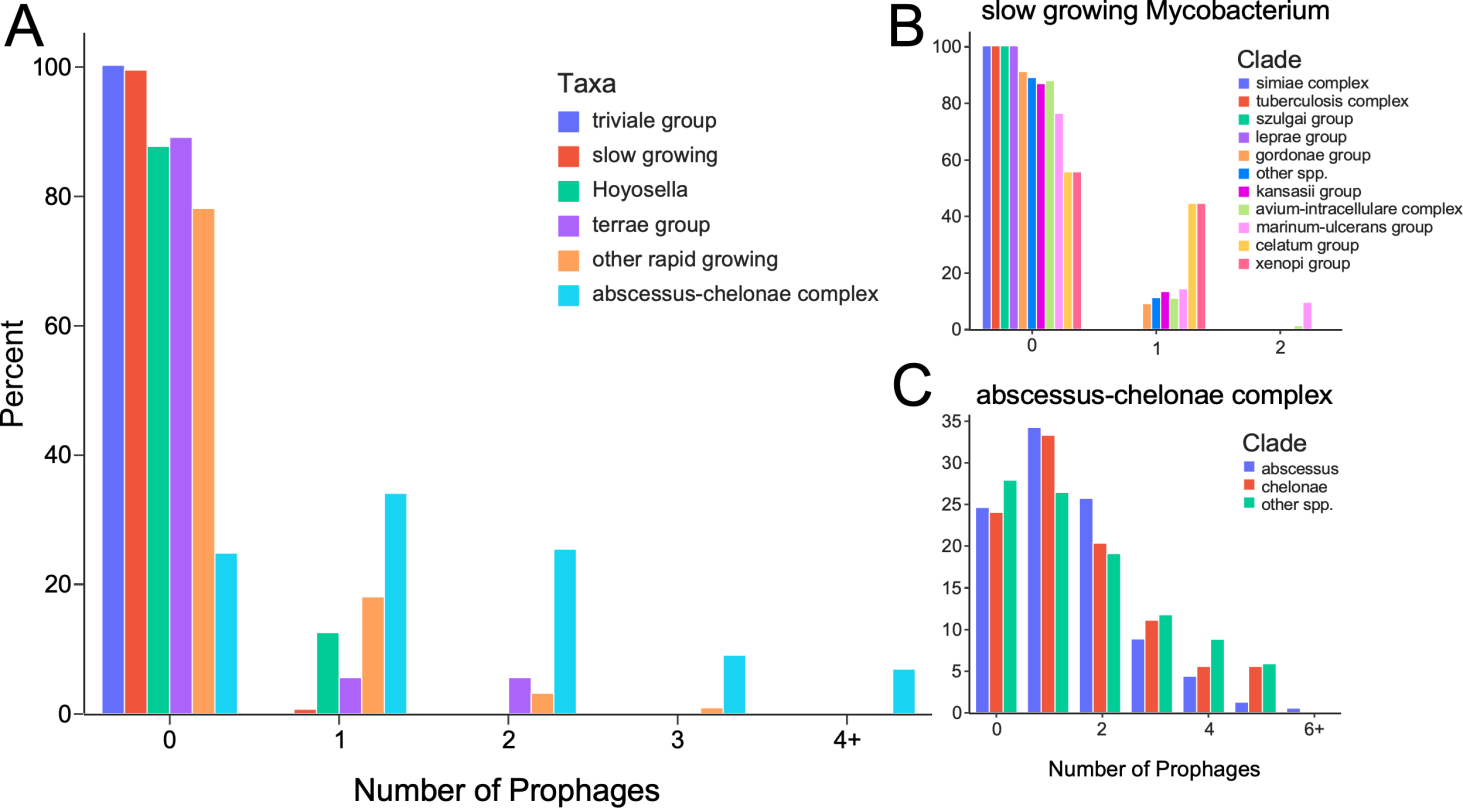
Discovery of *Mycobacterium* prophages. (**A**) Distribution of the number of prophages within strains from the major clades of *Mycobacterium* spp. as identified by DEPhT supplemented with SPLICE. (**B**) A detailed distribution of the prophage distribution in different groups of slow-growing *Mycobacterium*. (**C**) Species within the *M. abscessus-chelonae* complex.

The distribution of prophages in these strains is quite striking as it is highly variable among different taxa (Fig. 1A; Table 1); most strains within five major taxa are prophage free, whereas 75% of strains within the *M. abscessus-chelonae* complex contain at least one prophage (Fig. 1A). However, we note that *Mycobacterium* genome sequencing is highly skewed toward pathogenic strains, and the vast majority of sequences (~95%) belong to strains within the *M. abscessus-chelonae*, *M. avium-intracellulare* (MAI), or *M. tuberculosis* complexes. As such, these distributions should be contextualized with the heterogenous variations in the numbers of sequenced genomes within each of the major taxa. For example, fewer than 1% of slow-growing *Mycobacterium* carry a prophage, although ~94% of the sequenced genomes in this clade are *M. tuberculosis* genomes (Table 2) which, as reported previously, are prophage-free (30). Several other groups of slow-growing strains contain prophages, but the prophages are not present in the majority of sequenced genomes within those groups (Table 2; Fig. 1B). Of particular interest are those strains within the MAI complex because they are relatively common opportunistic pathogens and are often phage-resistant as well as multi-drug resistant. We identified more prophages in MAI strains than reported previously (28), although only 14.2% of the genomes carry a prophage. Given the overall paucity of prophages in these slow-growing strains, it is not surprising that strains rarely carry more than one prophage (Fig. 1B). In contrast, prophage-containing strains within the *M. abscessus-chelonae* complex often carry more than one prophage and are more likely to carry multiple prophages than none at all (Fig. 1C). Of all the strains examined, we identified at least one prophage in at least one genome of 44 defined species as well as 40 genomes that do not have species assignations (Table 3).

**TABLE 2:** Average number of prophages in slow-growing *Mycobacterium* subclades

Species group^*a*^	# genomes^*b*^	# genomes with prophage(s)^*c*^	# prophages^*d*^	Mean # prophages^*e*^
*M. simiae* complex	17	0	0	0
*M. tuberculosis* complex	6,620	0	0	0
*M. szulgai* group	13	0	0	0
*M. leprae* group	11	0	0	0
*M. gordonae* group	11	1	1	0.091
Other *Mycobacterium* spp.	134	15	15	0.112
*M. kansasii* group	30	4	4	0.133
*M. avium-intracellulare* complex	155	19	22	0.142
*M. marinum-ulcerans* group	21	5	7	0.333
*M. celatum* group	9	4	4	0.444
*M. xenopi* group	9	4	4	0.444
Totals^*f*^	7,030	52	57	0.006

^*a*^ Species group in the slow-growing *Mycobacterium* clade.

^*b*^ The total numbers of genomes from each group analyzed with DEPhT and SPLICE.

^*c*^ The total numbers of genomes from each group with one or more prophages identified using DEPhT and SPLICE.

^*d*^ The total numbers of intact prophages identified using DEPhT and SPLICE for each group of species.

^*e*^ The average numbers of prophages for each group of species.

^*f*^ The total numbers of genomes and prophages identified in slow-growing *Mycobacterium*.

**TABLE 3:** Prophage abundance in NTM species that carry prophages

Species^*a*^	# genomes^*b*^	# genomes ≥1 prophage^*c*^	# prophage^*d*^	Mean # prophages^*e*^	Unique # prophages^*f*^
*M. immunogenum*	17	17	61	3.588	52
*M. mucogenicum*	5	3	8	1.6	6
*M. chelonae*	54	41	85	1.574	71
*M. abscessus*	1,831	1,380	2,594	1.417	1,529
*M. franklinii*	12	6	13	1.083	9
*M. conceptionense*	6	6	6	1	5
*M. hassiacum*	3	3	3	1	1
*M. senegalense*	3	2	3	1	2
*M. branderi*	2	2	2	1	2
*M. heraklionensis*	2	1	2	1	2
*M. aichiense*	2	2	2	1	2
*M. fluoranthenivorans*	2	1	2	1	2
*M. sphagni*	2	1	2	1	2
*M. botniense*	1	1	1	1	1
*M. stephanolepidis*	1	1	1	1	1
*M. algericus*	1	1	1	1	1
*M. brisbanense*	1	1	1	1	1
*M. insubricum*	1	1	1	1	1
*M. pulveris*	1	1	1	1	1
*M. nivoides*	1	1	1	1	1
*M. persicum*	1	1	1	1	0
*M. litorale*	4	2	3	0.75	3
*M. peregrinum*	6	4	4	0.667	4
*M. saopaulense*	3	2	2	0.667	1
*M. thermoresistibile*	3	2	2	0.667	2
*M. xenopi*	5	3	3	0.6	2
*M. marinum*	12	1	7	0.583	6
*M. salmoniphilum*	10	4	5	0.5	4
*M. fortuitum*	8	4	4	0.5	4
*M. boenickei*	4	1	2	0.5	2
*Hoyosella altamirensis*	2	1	1	0.5	1
*M. europaeum*	2	1	1	0.5	1
*M. vulneris*	2	1	1	0.5	1
*M. dioxanotrophicus*	5	1	2	0.4	2
*M. celatum*	3	1	1	0.333	1
*M. shimoidei*	3	1	1	0.333	1
*M. austroafricanum*	3	1	1	0.333	1
*M. cosmeticum*	3	1	1	0.333	1
*M. septicum*	3	1	1	0.333	1
*M. avium*	68	13	16	0.235	9
*M. phlei*	5	1	1	0.2	0
*M. paragordonae*	5	1	1	0.2	1
*M. kansasii*	28	3	3	0.107	1
*M. intracellulare*	68	5	5	0.074	5
Other *Mycobacterium* ^*g*^	44	40	49	1.114	40
Subtotal	2,248	1,572	2,908	1.294	1,787
Total^*h*^	2,611	1,572	2,908	1.114	1,787

^*a*^ Species in the genus *Mycobacterium* and the closely related strain *Hoyosella altamirensis* for which at least one genome entry contains at least one prophage, sorted by the mean number of prophages found among the species.

^*b*^ The total numbers of genomes from each species analyzed with DEPhT and SPLICE.

^*c*^ The total numbers of genomes from each species with one or more prophages identified using DEPhT and SPLICE.

^*d*^ The total numbers of intact prophages identified using DEPhT and SPLICE for each bacterial species.

^*e*^ The average numbers of prophages for each species are shown.

^*f*^ The total numbers of prophages with unique sequences identified using DEPhT.

^*g*^ Strains in the genus *Mycobacterium* which are not speciated as labeled at PATRIC.

^*h*^ The total numbers of genomes, prophages, and unique prophages in nontuberculous bacteria. The total number of strains includes some which do not have species designations. Strains from species which have no predicted prophages are excluded from the subtotals.

Within the prophage-containing species, we identified a total of 1,572 individual bacterial genomes that contain at least one prophage (Table 3). The most highly represented species is *M. abscessus* and 1,380 of these genomes (from a total of 1,831) contain at least one prophage (Table 3). The total number of *M. abscessus* prophages identified in these is 2,594, with an average of 1.42 prophages per genome. However, many of the prophages are identical to each other and the number of unique (non-redundant) prophages we identified in *M. abscessus* is 1,529 (Table 3). For all strains tested here, we identified a total of 2,908 prophages, of which 1,787 are unique (Table 3).

### Genetic diversity of *Mycobacterium* prophages

The predicted *Mycobacterium* prophages were assembled with *M. smegmatis* phages to give a database of 3,308 genomes for comparative analysis using Phamerator (33). Phamerator assigns each gene to a Phamily (Pham) with a designator (Pham*Number*) using previously described methods (34). The genomes were then assorted into clusters of related genomes as described previously (15, 33 – 35). A total of 61 clusters were assembled and 18 genomes were classified as singletons as they currently have no close relatives (Table 4). Extant cluster designations used for the *M. smegmatis* phages were maintained, but the previously used prophage cluster designations (MabA, MabB, etc.) were subsumed into the *M. smegmatis* nomenclature (Table 4). For example, Cluster A now includes those prophages grouped in MabJ and this is recognized by the naming system Cluster A (+MabJ). Thirty new clusters (HA–ID) were assigned containing new and previously described prophages (Table 4).

**TABLE 4:** Mycobacterium phage and prophage clusters

Cluster^*a*^	# Phages	Mean length (bp)	Mean # CDS^*b*^	Mean # tRNAs^*c*^
A (+MabJ)	742	51,721	91	1
B	360	68,784	100	0
C	163	155,555	231	32
D	21	64,805	89	0
E	114	75,542	145	2
F	203	57,326	104	0
G	66	42,268	63	0
H	10	69,108	96	0
I	8	49,818	78	0
J	38	111,065	236	1
K	165	59,894	95	1
L	95	75,349	123	12
M (+MabI)	27	81,286	138	21
N	38	42,905	69	0
O	21	71,287	126	0
P	43	47,917	79	0
Q	20	53,849	84	0
R	8	71,339	100	0
S	17	64,993	113	0
T	7	42,746	62	0
U	3	69,864	108	1
V	4	77,869	148	20
W	8	61,079	90	1
X	2	88,037	177	4
Y	4	76,836	132	2
Z	2	50,807	86	0
AA	2	140,785	214	0
AB	6	49,359	72	0
AC	4	70,029	120	1
AD (+MabL)	53	65,842	91	0
AE	2	71,497	107	0
HA (MabA)	150	62,617	102	0
HB (MabB)	139	40,152	59	0
HC (MabC)	168	51,353	70	0
HD (MabD)	64	52,670	80	0
HE (MabE)	76	60,206	76	0
HF (MabF)	25	54,403	78	0
HG (MabG)	101	54,495	79	0
HH (MabH)	39	43,299	64	0
HI	6	49,318	69	0
HJ	5	51,843	72	0
HK (MabK)	66	75,368	115	0
HL	5	55,466	88	0
HM	4	54,297	80	0
HN (MabN)	52	41,488	65	0
HO (MabO)	21	45,419	67	0
HP (MabP)	5	51,658	62	0
HQ (MabQ)	16	75,860	106	0
HR (MabR)	13	67,456	96	0
HS	4	58,526	89	0
HT	4	78,035	133	3
HU	3	43,693	61	0
HV	2	38,330	56	1
HW	2	60,248	94	1
HX	2	50,134	82	0
HY	2	63,350	96	4
HZ	2	45,852	70	1
IA (MabA2-3)	44	61,507	102	0
IB	2	62,878	97	0
IC	3	54,210	77	0
ID	9	43,128	65	0
*DS6A*	N/A	60,588	97	0
*Kumao*	N/A	70,373	115	0
*LilSpotty*	N/A	49,798	85	0
*MooMoo*	N/A	55,178	97	0
*prophi1185650.276_36-1*	N/A	49,584	72	0
*prophi1214063.4_1-1*	N/A	56,097	78	0
*prophi146020.3_10-1*	N/A	50,110	75	0
*prophi1764.28_34-1*	N/A	56,684	69	0
*prophi1767.33_1-1*	N/A	79,626	131	27
*prophi2051551.3_3-1*	N/A	37,702	59	0
*prophi258505.4_5-1*	N/A	44,470	65	0
*prophi29313.6_5-1*	N/A	48,433	66	1
*prophi319705.112_2-1*	N/A	46,562	69	0
*prophi418031.3_7-1*	N/A	61,690	85	0
*prophi451644.8_76-1*	N/A	54,733	85	0
*prophi482462.3_2-2*	N/A	60,478	93	0
*prophi512402.5_9-1*	N/A	48,313	73	0
*prophiT5079-1*	N/A	55,420	80	0

^*a*^ Cluster designation, with previously published clusters indicated in parentheses; singleton phages are indicated in italics.

^*b*^ Average number of protein-coding genes for phages in the cluster.

^*c*^ Average number of tRNA genes for phages in the cluster.

**Fig 2:**
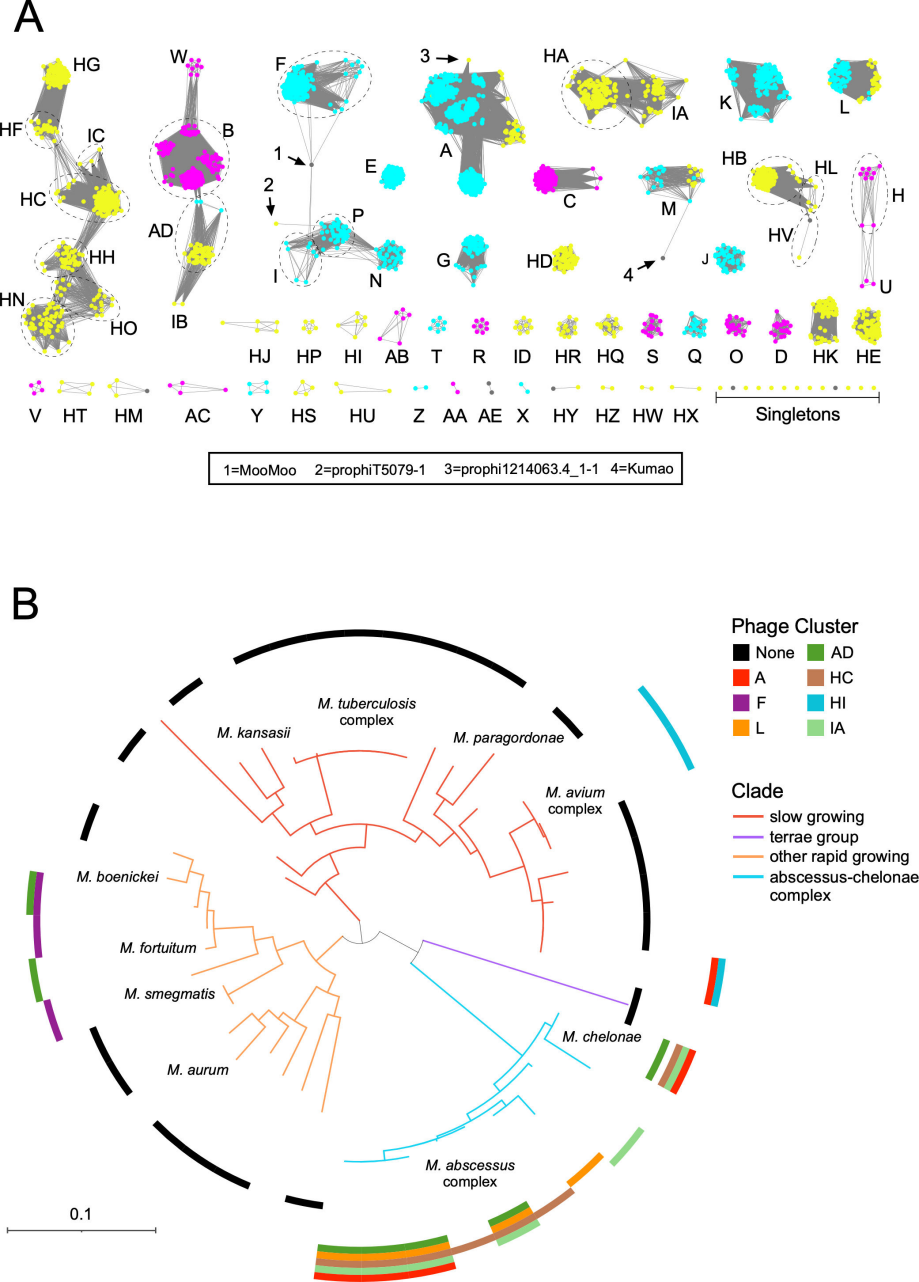
Relationships between *Mycobacterium* prophages. (**A**) A network of *Mycobacterium* phage and prophage genome relationships is shown, with nodes colored according to phage lifestyle (*M. smegmatis* lytic phages, magenta; *M. smegmatis* temperate phages, blue; lifestyle not known; gray); newly discovered *Mycobacterium* prophages are shown as yellow nodes. Groups of nodes within clusters are shown in dotted circles. Network construction used pairwise 25% proteome equivalent to join nodes. Four genomes of note (1,2, 3, and 4) are indicated. (**B**) A core genome phylogeny of representative genomes from PATRIC taxa with at least three members was constructed using RaxML. Branches of the phylogeny are colored according to their major clade in a similar scheme as in Fig. 1A. Colored arcs at the edges of the phylogeny indicate whether one or more members of select phage clusters can be found in the taxon, whereas a black arc indicates no prophages were found in that taxon.

A total of 11,602 protein Phams were identified through the pham assembly process, but only 1,180 (~10%) are shared by *M. smegmatis* phages and the *Mycobacterium* prophages, indicating that exchange between these phage/prophage populations is relatively uncommon. The *Mycobacterium* prophages and *M. smegmatis* phages thus generally assort into different clusters, although there is some overlap, primarily with temperate *M. smegmatis* phages as expected (Fig. 2A). For example, Clusters A, L, M, and AD each contain *M. smegmatis* phages and *Mycobacterium* prophages (Fig. 2A; Table 4), and three extant mycobacteriophage singletons (Sparky, MalagasyRose, and IdentityCrisis) join with prophages to form Clusters HY, HV, and HM. In one instance, there are genetic connections between lytic *M. smegmatis* phages (Cluster B) and Cluster AD prophages, although they “connect” through three temperate *M. smegmatis* Cluster AD phages (Fig. 2A). We also note that there is a complex network of *Mycobacterium* prophages spanning Clusters HG, HF, IC, HC, HH, HO, and HN (Fig. 2A). In each case, there are subsets of genomes with sufficient similarity to connect the nodes, suggesting they do undergo recombination with each other.

Consistent with this overall pattern is the finding that related prophages (i.e., within the same cluster) are commonly associated with bacteria within particular groups of bacterial hosts, and rarely extend across group boundaries (Fig. 2B). There are, however, some notable exceptions to this (Fig. 2B). One example of interest is the genomes in Cluster A, which is large and contains the largest number of subclusters. The newly constructed Cluster A contains over 700 *M*. *smegmatis* phages to which 30 *Mycobacterium* prophages have been added, including those formerly grouped in MabJ (Fig. 2B; Table 4). The Cluster A prophages are present in two major clades within the *M. abscessus-chelonae* complex but are also present in *Mycobacterium terrae* group (Fig. 2B). Similarly, Cluster AD prophages are present in three *M. abscessus-chelonae* group clades as well as in two clades of fast growers (Fig. 2B). These classes of prophages are of interest as they may have relatively broad host ranges and are thus of interest as potential therapeutic candidates if they can be grown lytically.

### Characterization of *M. abscessus* prophage integration sites

**Fig 3:**
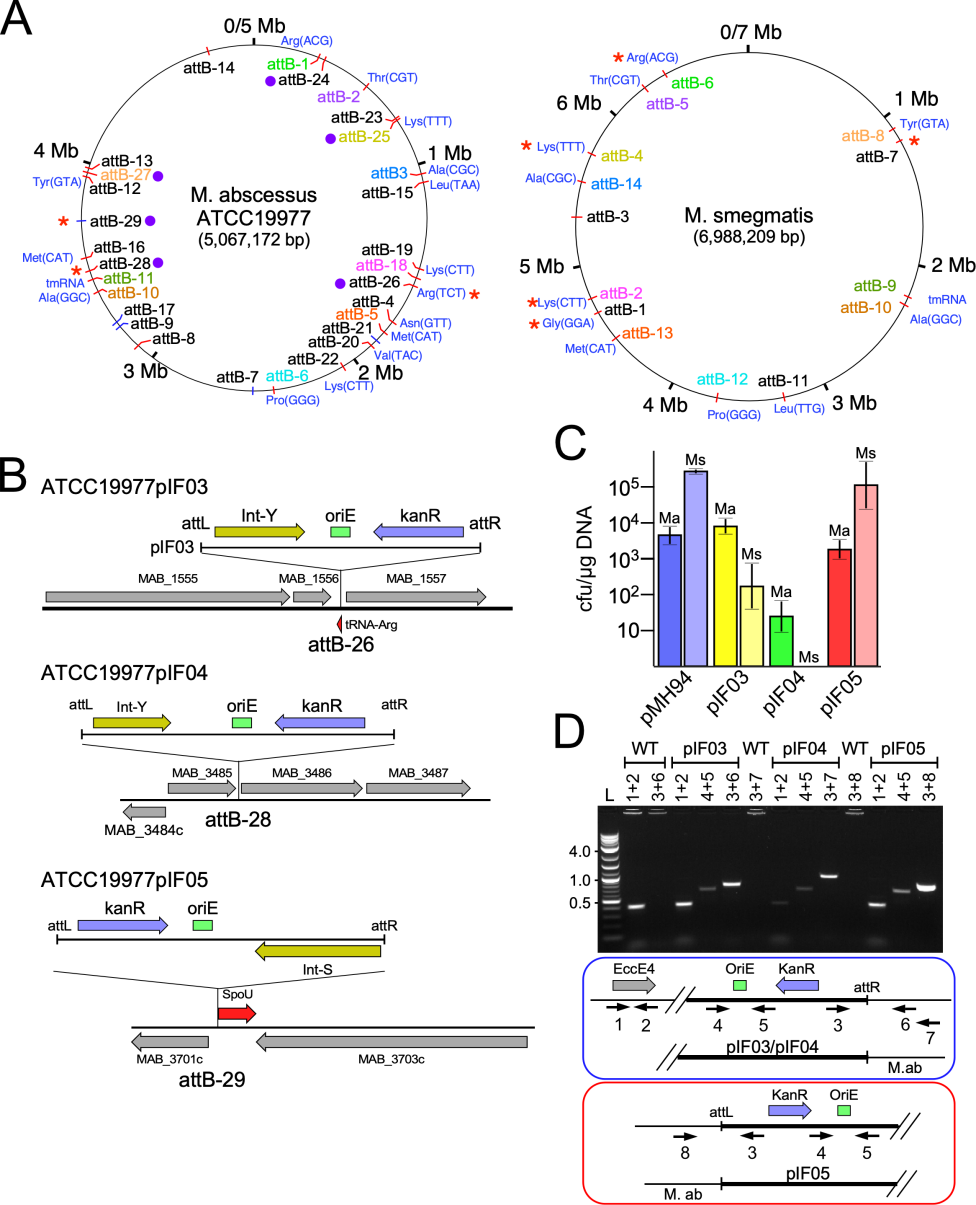
*attB* integration sites in *M. abscesses* and *M. smegmatis*. (**A**) The *attB* sites used for prophage integration on *M. abscessus* (left) and *M. smegmatis* (right) are shown. Although the *M. abscessus attB* sites were identified in many different strains, they are displayed on the *M. abscessus* ATCC19977 as a reference genome. All of the *M. smegmatis attB* sites identified are in *M. smegmatis* mc^2^155. The *attB* labels are colored to indicate analogs *attB* sites in *M. abscessus* sites and *M. smegmatis* (e.g., *M. abscessus* attB-1 is analogous *to M. smegmatis* attB-6). Red and blue tick marks on the circle indicate the *attB* sites used by Int-Y and Int-S site-specific recombination systems, respectively. Where the *attB* sites overlap host tRNA genes, the tRNA isotype and the anticodon are shown in blue type. Red asterisks indicate *attB* sites for which integration-proficient vectors have been developed. Purple circles denote newly discovered *M. abscessus attB* sites from this study. (**B**) Integration of three newly developed integration-proficient plasmids, pIF03, pIF04, and pIF05, derived from prophages prophi1962118.185_4-1, prophi319705.109_14-4, and prophi1185650.948_10-1, respectively. The *attB* sites are shown with their surrounding genes designated as in the ATCC19977 genome with the integration vector shown above in the integrated form, showing the kanR gene, oriE, and either a tyrosine integrase or serine integrase. The attB-26 site overlaps a tRNA-arg gene, attB-28 is intergenic between MAB_3485 and MAB_3486, and attB-29 overlaps the translation start site of the tRNA-methyltransferase, SpoT. (**C**) Transformation efficiencies (CFU/μg) of pMH94 (38), pIF03, pIF04, and pIF05 into *M. smegmatis* mc^2^155 *M. abscessus* ATCC19977. The average transformation efficiency for pMH94 (blue), pIF03 (yellow), pIF04 (green), and pIF05 (red) is displayed, with standard error. Transformation of *M. abscessus* is labeled as Ma and displayed in vibrant colors while transformation of *M. smegmatis* is labeled as Ms and displayed in pastel colors. (**D**) PCR verification of *M. abscessus* ATCC19977 transformants of pIF03, pIF04, and pIF05. For transformants of *M. abscessus*, PCR reactions were designed to amplify a conserved gene in *M. abscessus* (primers 1 + 2), a portion of the vector shared between all plasmids (primers 4 + 5), and the junction created by integration of the specific vector (primers 3 + 6,7,8). A schematic of the designed amplification reactions is displayed below the gel.

Prophages are commonly formed by integrase-mediated site-specific recombination of temperate phage genomes, and all of the previously described chromosomally integrated *Mycobacterium* prophages are predicted to have been formed in this way. Most of those prophages identified using DEPhT are found in strains of the *M. abscessus-chelonae* complex and encode site-specific integration systems. Using this large collection of prophages in *M. abscessus*, we determined the repertoire of *attB* sites used for integration, although only 35% of the identified prophages had a readily identifiable *attB* site; many of the others use serine integrases for which the attachment sites with short common core sequences are not easily predicted. Previously, 23 *M*. *abscessus attB* sites have been described (29, 36, 37), and we identified here an additional six sites (attB-24–attB-29; Fig. 3A; Table S2). Of the 29 *attB* sites, 23 use tyrosine integrases (Int-Y) and six use serine integrase (Int-S) site-specific recombination systems. Sixteen of the Int-Y systems use *attB* sites overlapping host tRNA genes or the tmRNA gene (Fig. 3A; Table S2), but seven do not.

The *attB* sites are generally distributed around the *M. abscessus* genome, with the notable exception of a ~1 Mbp region between the 4 Mbp and 5 Mbp positions where only a single *attB* is located (attB-14, Fig. 3A). The dearth of *attB* sites in this region likely results at least in part because of the paucity of tRNA genes, with only a single tRNA^Gly^ at coordinate 4,394,540; no prophage integration events have been identified at that location (Fig. 3A). In total, 15 of the 47 tRNA genes (corresponding to the ATCC19977 strain) are used as sites for phage integration (Fig. 3A). For other *Mycobacterium* clades, we were able to predict *attB* sites for 67% of *M. marinum-ulcerans* group prophages, 31% of *M. avium-intracellulare* complex prophages, and 25% of *M. fortuitum-smegmatis* group prophages. All of these *attB* sites overlap tRNA^Lys^-(TTT) or tmRNA genes.

The number of *attB* sites used by *M. abscessus* prophages is more than twice the number identified for temperate phages in *M. smegmatis* (Fig. 3A). Of the newly identified *M. abscessus attB* sites in this study, only two (attB-25 and attB-27) have analogous sites in *M. smegmatis,* the other four do not (Fig. 3A). To test whether these integration systems recombine at their predicted *attB* sites, we constructed integration-proficient plasmid vectors (38 – 40) to integrate into attB-26, attB-28, and attB-29 (pIF03, pIF04, pIF05, respectively), carrying the predicted *attP* and *int* gene sequences from prophages integrated at those sites (Fig. 3B; Table S3). Two of these use tyrosine integrase systems and one (pIF05) uses a serine integrase. Each plasmid was transformed into *M. abscessus* ATCC19977, which has unoccupied *attB* sites for all three plasmid vectors; we also transformed these into *M. smegmatis* mc^2^155 (Fig. 3C). Plasmids pIF03 and pIF05 transformed *M. abscess*us with similar efficiencies as integration vector pMH94 (38), and pIF04 at a lower efficiency (Fig. 3C). PCR analysis of *M. abscessus* transformants confirmed that these plasmids integrated into the predicted *attB* sites (Fig. 3D; Table S3). Plasmids pIF03 and pIF04 did not efficiently transform *M. smegmatis*, which presumably lacks analogous *attB* sites for these (Fig. 3A and B). Surprisingly, pIF05 transformed *M. smegmatis* with a similar frequency to pMH94, even though it does not have an analogous *attB* site, suggesting it may be somewhat promiscuous in its target site choice (Fig. 3C). The pIF05 plasmid is predicted to integrate into the sixth codon of an SpoU-like tRNA methyltransferase (MAB_3702) in *M. abscessus*; it is unclear if expression is reconstituted following integration (Fig. 3B), but the gene is non-essential for growth (41). Plasmid pIF04 integrates in the intergenic region between genes MAB_3485 and MAB_3486; both are predicted to be non-essential for growth (41). Integration of pIF03 is predicted to reconstitute the host tRNA^Arg^ gene.

### Phage-encoded ESX-secreted toxin (PEST) cassettes

**Fig 4:**
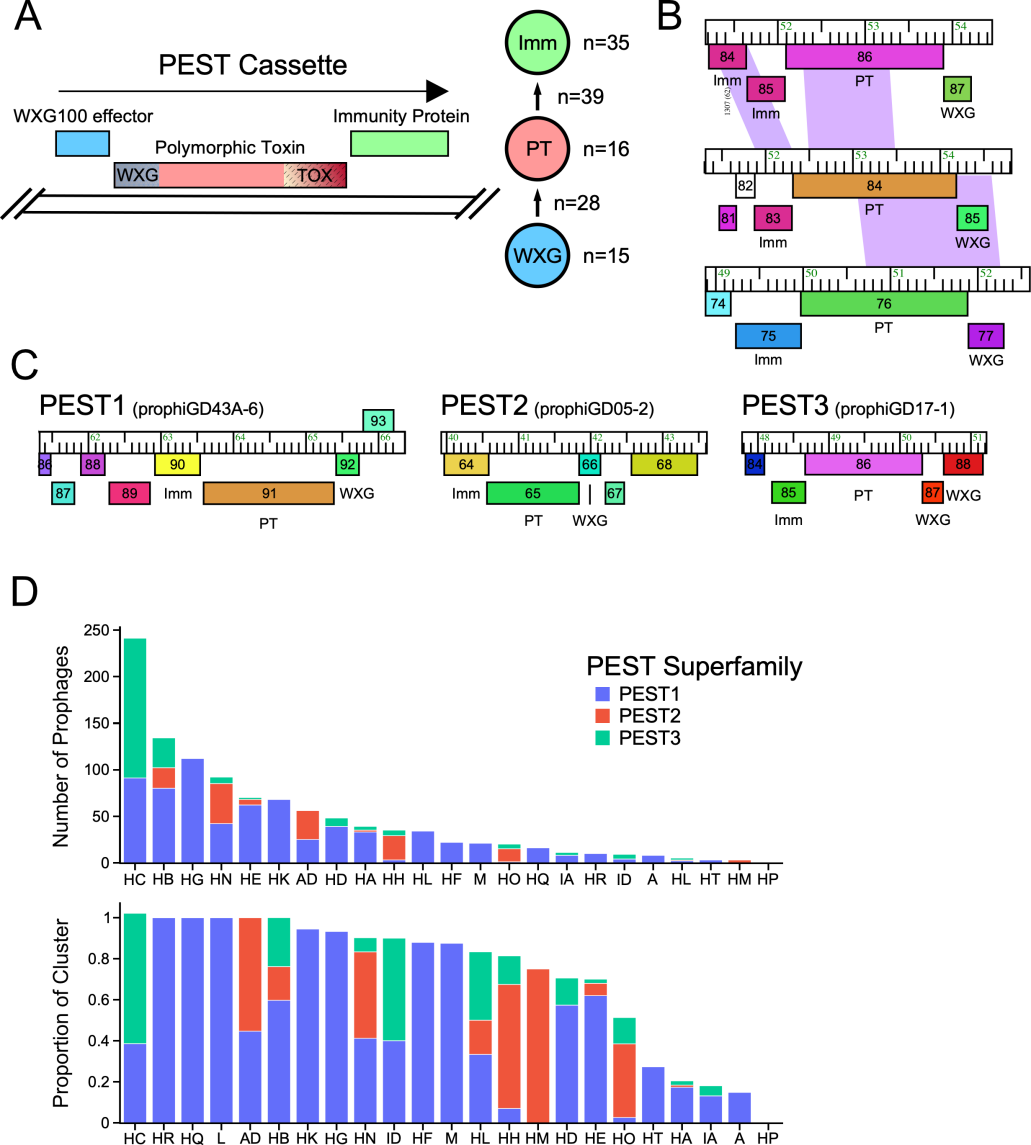
PEST cassettes in *Mycobacterium* prophages. (**A**) PEST cassettes typically include three genes encoding a small (~100 amino acid) WXG100 effector protein, a PT, and an Imm. Many combinations of genes are known, and the large toxin proteins contain several domains in different arrangements, including an N-terminal WXG100-like domain (WXG) and a variety of different C-terminal domain toxin domains (TOX). The immunity protein typically co-evolves with the TOX domain, and the N-terminal WXG100 domain is predicted to form a heterodimer with the WXG100 effector protein. To the right is a graph showing the numbers of distinct sequence groups for the WXG, PT proteins, and Imm proteins, and the numbers of pairwise combinations of WXG:PT and PT:Imm genes are shown next to the arrows. The full graph of the relationships is shown in Fig. S1. (**B**) PEST cassettes often recombine with other PEST systems. Genome maps of PEST systems prophiGD12-2, prophiGD03-1, and prophiGD08-3 (top to bottom) illustrate nucleotide similarities (purple) and likely recombination events between both the secretion signal and TOX domains. (**C**) PEST systems can be assorted into superfamilies based on remote sequence homology. HMM-HMM profile searches between phams of polymorphic toxins yield three major PEST superfamilies with PT proteins that share N-terminal homology, as illustrated in Fig. S1. Genome maps of representative PEST systems from each of the superfamilies are displayed. Cassettes encoded by prophiGD43A-6 and prophiGD05-2 of PEST1 and PEST2 superfamilies, respectively, are organized in a three-gene cassette of WXG, PT, and Imm. PEST3 systems, and the representative encoded by prophiGD17-1, are organized into a four-gene cassette, with two WXG100 effector genes. (**D**) PEST cassettes are unevenly distributed among prophage clusters. Graphs display counts (top) and proportions of prophage clusters of unique prophages that encode PEST cassettes. Counts and proportions are divided and colored based on the superfamilies of the PEST systems, with PEST1, PEST2, and PEST3 in blue, red, and green, respectively.

We noted previously (29) that a common feature of *M. abscessus* prophages is a three-gene cassette coding for a polymorphic toxin-like protein (PT), an immunity protein (Imm), and a WXG100 ESAT6-like protein (Fig. 4A); a similar system has been described in *M. chelonae* McProf and other MabR prophages (37, 42). The polymorphic toxin proteins typically have a C-terminal domain associated with cellular toxicity, and an N-terminal domain similar to WXG100 motifs (Fig. 4A). Because WXG100 proteins are typically secreted via a Type VII secretion system, we will refer to these as PEST systems (Fig. 4A). PEST systems are lysogenically expressed from at least some prophages (29), although their roles in *M. abscessus* physiology are unclear.

To comprehensively identify PEST systems in *Mycobacterium* prophages, we employed a two-step approach. First, homology searches were used to identify those PEST components that are related to previously described PEST systems (29, 37). Secondly, PEST systems lacking evident sequence similarity to previously described PESTs were identified using an iterative gene neighborhood network analysis (Fig. S1). This identified 35, 16, and 15 distinct sequence types of Imm proteins, PT proteins and WXG protein, respectively, but in a multitude of different combinations (Fig. 4A), with evidence of recombination both between and within PEST component genes (Fig. 4B).

Remote sequence similarity searches between phams of polymorphic toxins reveal three distinct and conserved N-terminal domains and can be grouped into superfamilies based on this domain, designated PEST1, PEST2, and PEST3 (Fig. 4C). These PT proteins are generally associated with specific subsets of WXG100 effector proteins, and likely differ in how they are secreted by Type VII secretion systems, while different Imm proteins tend to co-distribute with particular PT C-terminal TOX domains (Fig. S1). Combinations of WXG-WXG100 effector proteins and TOX-Imm proteins are generally limited to one PEST superfamily (Fig. S1B), although there is one example of recombination between PEST cassettes of different PEST superfamilies (Fig. 4B). The PEST superfamilies are also distributed heterogeneously among prophages in different clusters (Fig. 4D). For example, there are no PEST2 systems in the abundant Cluster HC prophages (Fig. 4D), but they represent 75% of the systems in Cluster HH prophages. Likewise, PEST3 systems are the majority in Cluster HC, but represent only about 20% of Cluster HB prophages (Fig. 4D). PEST systems are sparse in prophages of some clusters, including Clusters A, IA, HT, HA, and HP (Fig. 4D). Interestingly, of the 1,063 unique prophages with PEST systems we have identified here, all are in strains of the *M. abscessus-chelonae* complex, with only one exception in an *M. boenickei* prophage. Three proteins likely to be ESX secreted were found in *M. smegmatis* phages, Dori (gp88-89), Che9c (gp35-36), and Sbash (gp35-36), although none contain all the constituent components of PEST systems.

**Fig 5:**
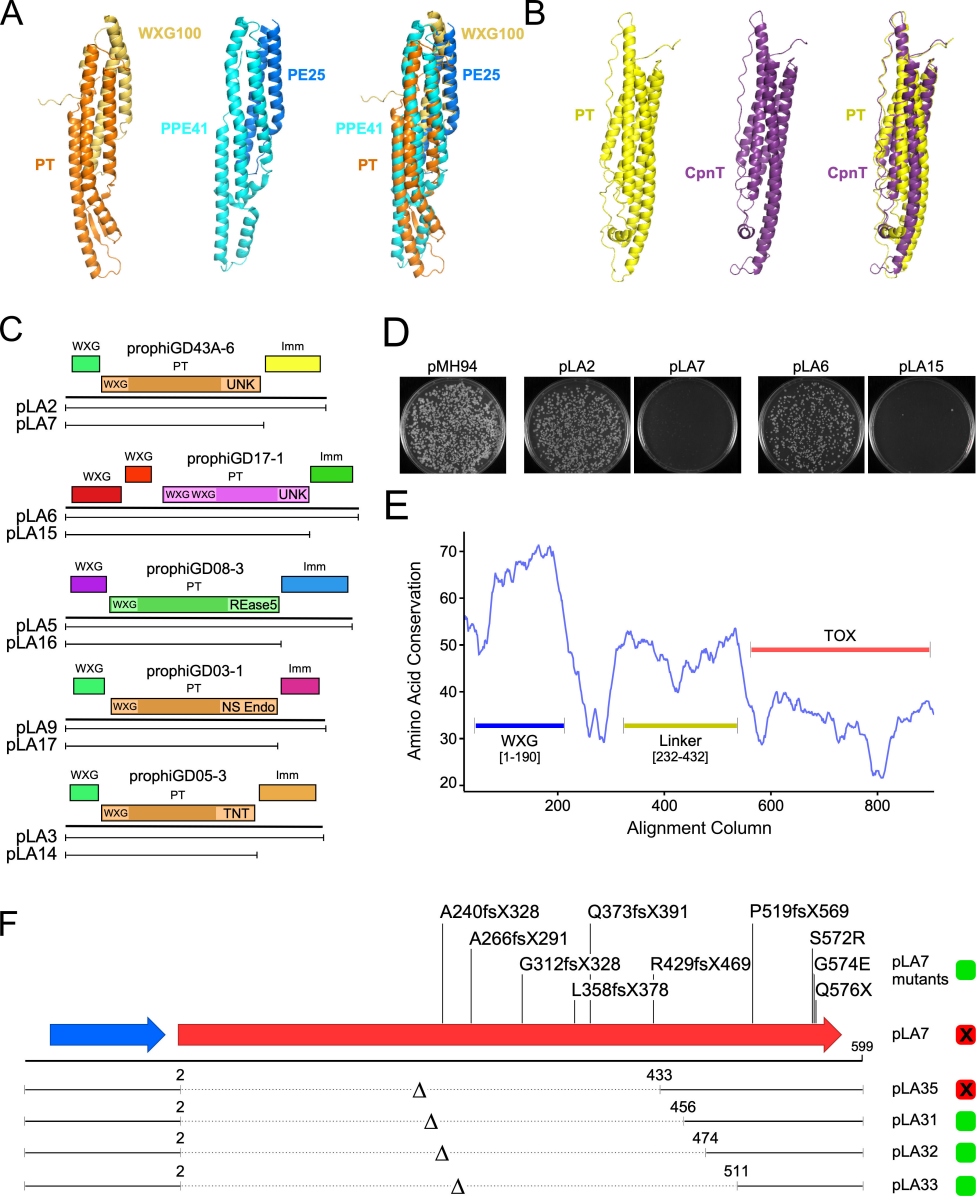
Characterization of PEST systems. (**A**) At the left is shown the AlphaFold-predicted structure of a heterodimer formed by the prophiGD43A-6 PT N-terminal secretion signal domain (residues 1–173) (orange) and the WXG100 effector protein (yellow; gp92 and gp91, respectively). In the center is the structure of the secretion domains of the PE25/PPE41 complex (RSCB PDB 2G38) (43) shown in blue and light blue, respectively (45); at the right is shown a superimposition of the two structures. (**B**) At the left is the AlphaFold prediction of the N-terminal domain (residues 1–310) of the prophiGD17-1 PT protein (gp86; yellow), in the center is the AlphaFold prediction of the secretion domain of *M. tuberculosis* CpnT (purple) (44). The two structures are superimposed at the right. (**C**) PEST systems from five prophages are shown, with the segments cloned (with or without the *imm* genes) into integration vectors, with the plasmid names at the left. (**D**) Recombinant plasmids with PEST cassettes were transformed into *M. smegmatis*, and transformants recovered on selective media. Plasmids pLA7 and pLA15 that lack the cognate *imm* genes to not efficiently transform consistent with toxicity conferred by the PT proteins. (**E**) Amino acid conservation in polymorphic toxins related to prophiGD43A-6 gp92 is plotted to reveal the domain boundaries between the WXG, linker, and TOX domains. (**F**) The organization of the WXG100 effector protein (blue) and polymorphic toxin (red) of prophiGD43A-6 are shown, with segments inserted into integration vectors shown below; the regions of the PT gene removed are shown (Δ), with the amino acid coordinates of the deletion boundaries indicated. The abilities of the plasmids to efficiently transform *M. smegmatis* is shown at the right (green positive, red negative). The positions of amino acid substitutions of non-toxic mutants are shown above the PT open reading frame (ORF). Amino acid substitutions are shown as the wild-type residue, codon number, and substituting amino acid (or stop codon, X). Frameshift mutations are shown as the wild-type residue followed by the codon with the mutation followed by the position of the first translation termination codon (X) encountered.

The PEST1 and PEST2 superfamilies are predicted to be secreted similarly to PE/PPE proteins, forming heterodimers between the WXG100 effector protein and the PT N-terminal WXG domain (Fig. 5A). AlphaFold-predicted structures of the PEST1 prophiGD43A-6 effector protein and the N-terminal domain (residues 1–173) of its PT protein (gp92 and gp91, respectively) form a heterodimer with evident structural similarity (root mean square deviation [RMSD] 3.7Å) to the heterodimer of the *M. tuberculosis* PE25/PPE41 complex (RSCB PDB 2G38) (43) (Fig. 5A). In contrast, PEST3 systems contain PT proteins with a longer N-terminal domain (~300 aa) with two WXG100 motifs that fold to form a four-helix bundle similar to the PEST1 heterodimer; this is reminiscent of *M. tuberculosis* CpnT (44) and the predicted structure of the prophiGD17-1 domain and CpnT domain are superimposable (RMSD 1.9Å) (Fig. 5B). These PEST3 systems also have two upstream genes encoding WXG100 effectors (Fig. 4C) analogous to the *esxF* and *esxE* genes upstream of *cpnT* (44).

To further characterize these PEST systems, we constructed recombinant plasmids (pLA2 and pLA6) containing the complete PEST systems from prophages prophiGD43-6 and prophiGD17-1 and their native expression signals (Fig. 5C). Both respective PT proteins contain predicted N-terminal WXG100 domains (one in the prophiGD43-6 PEST1 system, and two in the prophiGD17-1 PEST3 system; Fig. 5C), but neither has a bioinformatically informed C-terminal toxin domain, suggesting that these may have novel toxin activities. We also constructed plasmid derivatives of these (pLA7 and pLA15, respectively), lacking the predicted immunity proteins. These were then transformed into *M. smegmatis* to test for toxicity (Fig. 5D). We observed that plasmids pLA2 and pLA6 both efficiently transformed *M. smegmatis*, but plasmids pLA7 and pLA15 did not (Fig. 5C). These data suggest that the PT proteins can confer a toxic phenotype, and that the immunity proteins protect from this toxicity.

The PEST1 system of prophiGD43A-6 includes a PT with an N-terminal WXG100 domain, but the C-terminal domain with potential toxicity is ill-defined, although there is a short (~30 aa) region with some similarity to Ntox15 domains. To further characterize this region and other TOX domains lacking sequence similarity to characterized proteins, we examined regions of PT proteins for sequence conservation to delimit potential domain boundaries (Fig. 5E). These data suggest the likely location of the N-terminal WXG domain, a central linker domain, and a TOX domain positioned in the C-terminal 150–250 residues. We thus constructed a derivative of pLA7 (pLA35) containing just the C-terminal 166 residues of the prophiGD43A-6 PT and observed that this also confers the non-transformable phenotype (Fig. 5F). However, larger deletions, including removal of another 23 residues causes loss of toxicity (Fig. 5F). Finally, we mutagenized plasmid pLA7 by propagation in *Escherichia coli* XL1-red and selected non-toxic mutants by transformation into *M. smegmatis*. Fourteen mutants were sequenced and yielded 10 unique genotypes which lack toxicity, seven of which have frameshift mutations. One has a nonsense mutation (Q576X) that removes the most C-terminal 23 residues and two contain single amino acid substitutions, S572R and G574E, suggesting these are critical residues for the toxic function.

### Prophage-like elements and defective prophages in *Mycobacterium* genomes

**Fig 6:**
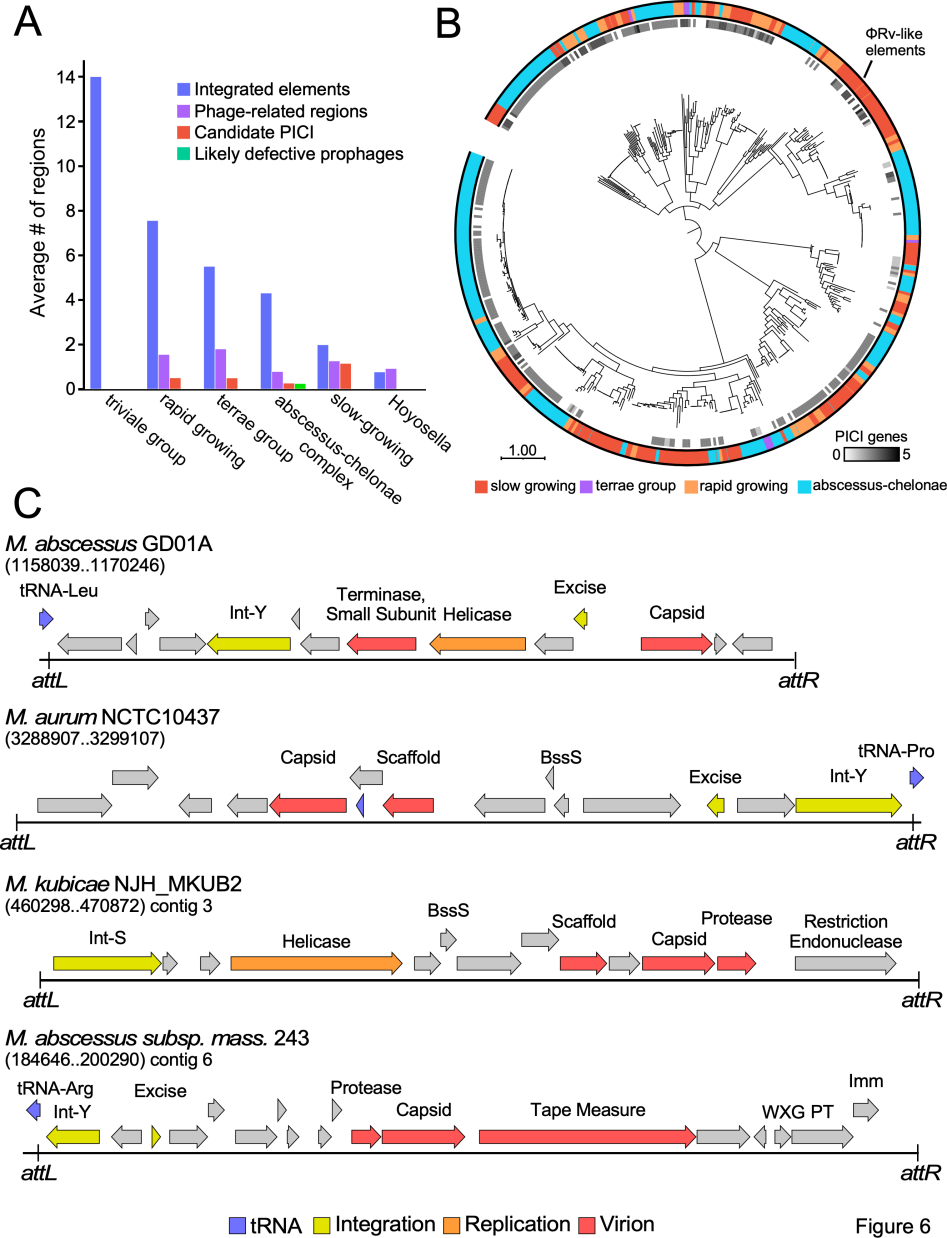
Prophage-like regions in *Mycobacterium* genomes. (**A**) The average numbers of integrated elements and phage-like regions in each clade of *Mycobacterium* are shown. Integrated elements (blue bars) were identified as genomic regions containing either an integrase or transpose, excluding prophages. Phage-related regions (purple bars) were identified as those containing one or more phage-like genes, regardless of whether there is a closely linked integrase/transposase. A subset of phage-related regions has both an integrase and a major capsid protein and was identified as candidate PICIs (red bars). Some of the phage-related regions have nucleotide sequence similarity to known phages or prophages (green bars). (**B**) A phylogeny of candidate PICIs based on the amino acid sequence of the major capsid proteins is shown at the center. The surrounding inner ring shows which of these are discovered as PICIs using SatelliteFinder (46), displayed as a grayscale; black indicates SatelliteFinder identified a PICI with all core genes, shades of gray indicate PICIs with missing core elements with lighter shades denoting PICIs with fewer core genes, and white indicates that SatelliteFinder did not identify the region as a PICI. The clade of *Mycobacterium* containing candidate PICIs is displayed in the outer ring, with slow-growing *Mycobacterium*, *Mycobacterium* related to *M. terrae*, rapidly growing *Mycobacterium*, and *M. abscessus-chelonae complex* bacterium shown in red, purple, orange, and cyan, respectively. (**C**) Genome maps of four representative candidate PICIs. The strains names and PICI coordinates are shown, and the attachment junctions *attL* and *attR* at the PICI boundaries are indicated. The three PICIs encoding tyrosine family integrases (Int-Y) integrate at tRNA genes, whereas the PICI encoding a serine family integrase (Int-S) does not. Phage genes coding for integration/excision, virion structural proteins, DNA replication functions, and tRNAs are colored yellow, red, orange, and blue, respectively. All four PICIs shown were identified here, but are not discovered by SatelliteFinder.

The prophages described above are likely to be mostly intact and capable of forming infectious phage particles. This is supported by recovery of several lytically propagated prophages (19, 29) and detection of viral particles by PCR in culture supernatants (36). To explore whether there are other integrated genomic elements and satellite regions, we searched first for genomic regions containing at least one homolog of either integrases/transposases (Fig. 6A; Fig. S2). Such regions are relatively common in several *Mycobacterium* clades with an average of 14/genome in the triviale group, 8/genome in other rapidly growing mycobacteria, 4/genome in the *M. abscessus-chelonae* group, and 2/genome in the slow-growing *Mycobacterium* group (Fig. 6A). However, only a small subset (Fig. 6A, green bars) have nucleotide similarity to known phages or prophages (defined as “defective prophages”; Fig. S1A and B; Data set S3), although interestingly, 87% of these contain PEST systems. However, many of the integrated elements lack other phage-related genes and are not evidently defective prophages (Fig. S1A). We also searched for genomic regions containing at least one homolog to a known phage protein, regardless of whether an integrase-like gene is closely linked to it (“phage-related regions”; Fig. 6A; Fig. S1B); these regions are also relatively uncommon and are present, on average, at fewer than two per genome (Fig. 6A, purple bars).

We then searched for regions containing both an integrase and a phage capsid gene, using the large collection of diverse capsid genes identified in *Mycobacterium* phages and prophages (Data set S4). It has previously been reported that *Mycobacterium* strains carry PICIs, and a subset (62%) of the regions we identified (Fig. 6A and B) is also identified as PICIs using SatelliteFinder (46). The phylogeny of the capsid genes shows that they are highly diverse, and SatelliteFinder may not have recognized some PICIs due to incomplete capsid representation (Fig. 6B). In general, candidate *Mycobacterium* PICIs with closely related capsid genes are in related *Mycobacterium* strains (Fig. 6B), but there are several instances of elements from different *Mycobacterium* clades with closely related capsid genes which are otherwise dissimilar, suggesting either horizontal gene transfer or convergent evolution between PICIs (Fig. 6B). Four examples of the newly identified PICIs (Fig. 6C) illustrate their general features. Some have tyrosine family integrases and integrate into host tRNA genes, with some of the *attB* sites corresponding to those used by prophages, including the *M. abscessus* GD01 PICI that uses attB-15 (Fig. 3 and 6); others use serine family integrases and integrate elsewhere (Fig. 6C). Some of the PICIs include DNA replication proteins such as DNA helicases, and other virion protein genes, including terminase and capsid assembly scaffold protein genes (Fig. 6A). Interestingly, 22% of the candidate PICIs we have identified also code for PEST systems (including *M. abscessus* subsp. *massiliense* strain 243, Fig. 6C). A phylogeny of the PICI capsid proteins together with phage and prophage capsid shows several instances of relationships that may correlate with the ability of some phages to induce replication of particular PICIs (Fig. S1C), although this has not yet been demonstrated experimentally. We note that in the slow-growing mycobacteria, these PICI-like elements are quite prevalent (Fig. 6A), and correspond to the previously described ϕRv1 and ϕRv2 elements (47 – 49), of which one or both are present in *M. tuberculosis* strains and sometimes present in other strains within the *M. tuberculosis* complex (Data set S5). PICIs with closely related capsid genes to ϕRv elements are present in some non-*M. tuberculosis* strains, again suggesting these are mobilizable.

## DISCUSSION

Bioinformatic characterization of *Mycobacterium* genomes reveals a heterogenous spectrum of genomic elements that constitute substantial parts of the accessory genome components and vary among *Mycobacterium* strains. This includes a large number of prophages that are likely intact and capable of induction and lytic growth. However, the distribution of these prophages is heterogenous among *Mycobacterium* strains. At one end of the spectrum, all sequenced *M. tuberculosis* strains are devoid of intact prophages, and at the other end, 75% of *M. abscessus* strains contain at least one prophage. In other *Mycobacterium* strains, prophage prevalence varies greatly although the numbers of sequenced genomes are far fewer than for *M. tuberculosis* or *M. abscessus*. The prophage prevalence may reflect the environments in which these various strains generally grow and the phages within those environments. The lack of prophages in *M. tuberculosis* remains a curiosity, as many phages have been identified that infect *M. tuberculosis*, many of which are temperate (18). However, there is no environmental reservoir of *M. tuberculosis* outside of humans, and phage *M. tuberculosis* encounters are presumably rare. In contrast, *M. abscessus* is commonly found in numerous environments (50). Most *M. tuberculosis* strains do, however, carry one or both of the PICI-like elements ϕRv1 and ϕRv2 (47). We also note that *M. abscessus* is Clustered Regularly Interspaced Short Palindromic Repeats (CRISPR)-free, whereas *M. tuberculosis* contains a CRISPR-Cas system that is reported to be at least partially active (51), and these may influence prophage profiles.

In general, the *Mycobacterium* prophages are genomically distinct from the *M. smegmatis* phages isolated as plaque-forming viruses, but there are several instances of the prophages and phages being sufficiently similar to be grouped together in the same clusters. These include the extant Clusters A, M, L, and AD, as well as the newly formed Clusters, HY, HV, and HM (Table 4). We anticipate that additional clusters containing both prophages and phage genomes will be formed as new genomes of both are sequenced. We note that no prophage relatives were identified of either Cluster G or Cluster K phages, which are generally temperate and several can efficiently infect some *M. abscessus* strains (19). We identified one prophage in strain *Mycobacterium* sp. MHSD3 (Table S1) that shares partial nucleotide sequence similarity with DS6A, whose host range is constrained to the *M. tuberculosis* complex (52). This may reflect environmental or geographical constraints, but we also note that most sequenced *Mycobacterium* strains are clinical isolates, and it is plausible that environmental isolates could have somewhat different prophage profiles.

The *M. abscessus* prophages integrate into a wide array of chromosomal attachment sites distributed around the genome, and not evidently localized toward the replication origin or terminus. The broad range of *attB* sites suggests that the use of these is actively evolved, conceivably driven by competition between phages for use of these sites. There are no regions of the genomes evidently excluded from integration, other than regions where tRNA genes are relatively rare (Fig. 3). Mapping these integration sites can facilitate the development of integration vectors for construction of recombinant strains, and having a large repertoire of such vectors can help avoid the limitations of finding the *attB* sites in any particular *M. abscessus* strain being occupied by a resident prophage. We demonstrate that three of the new integration systems are active and described three new integration proficient vectors for use in *M. abscessus* genetics.

*M. abscessus* strains carry numerous examples of ESX-secreted polymorphic toxins. These are prominent in the intact prophages described here (i.e., the PEST systems), but are also prevalent in defective prophages and in candidate PICI elements. The PEST systems are highly variable and can be sorted into three major groups (PEST1, PEST2, and PEST3) based on their ESX secretion motifs, and there is a large number of different toxin domains at the C-termini of the polymorphic toxins. However, the roles of these in *M. abscessus* physiology are unclear. They could be involved in pathogenicity and influence intracellular behavior in eukaryotic cells similar to *M. tuberculosis* CpnT (44), they could act in bacterial competition (53), or they could confer prophage-mediated viral defense (6). There is no direct support for any of these, although several systems are known to be lysogenically expressed (29, 42), and they are typically closely linked to the viral integration loci, properties of other known phage defense systems (29).

Finally, we note that the discovery of the prophages considerably expands the known diversity of *Mycobacterium* phage and prophage sequences, which in turn facilitates identification of new phage-like elements. For example, it enables the identification of an expanded set of candidate PICI elements, beyond those previously described (46). It remains to be determined if these are indeed induced and transduced by *Mycobacterium* phages.

## MATERIALS AND METHODS

### Discovery and extraction of prophages in *Mycobacterium* genome sequences

DEPhT (30) was used to discover and extract prophages for all complete genome sequences of *Mycobacteriaceae* strains, and whole-genome sequences of *Mycobacteriaceae* strains assembled into 100 or fewer contigs were retrieved from databases at PATRIC (54). DEPhT was run using the *Mycobacterium* model available in the DEPhT repository at the Open Science Framework (https://osf.io/zt4n3/) using default parameters. In addition, we developed related software referred to as SPLICE that assists DEPhT in identifying prophages spanning unjoined contigs. In brief, SPLICE uses a BLAST database of previously identified and clustered phage genomes as a scaffold to identify phage-like sequence fragments at the ends of contigs. Sequence fragments are ordered based on an alignment to a reference prophage or phage genome and concatenated to form a single sequence. Spliced fragments are assigned a cluster based on their reference phage genome and subsequently validated as a prophage with DEPhT. Code and further details for SPLICE is available at the github repository (http://github.com/laa89/SPLICE).

### Collection and annotation of *Mycobacterium* prophages

Prophage sequences produced by DEPhT were used to construct a MySQL database, under100contigMycos_v5 using *pdm_utils* and is publicly available at http://databases.hatfull.org/Published/. Identified prophages which were either truncated by contig ends or contained excess bacterial sequence were omitted from further analysis. Prophage genomes were annotated in a high-throughput manner by first clustering predicted protein products by their amino acid sequences with *pdm_utils* (55) and PhaMMseqs (34) and then assigning functional annotations to protein clusters based on sequence homology to existing sequence clusters and databases available at Hhpred (56). *Mycobacterium* phage and prophage genomes assembled into clusters and subclusters using PhamClust (35), and the resulting analyses were saved in a MySQL database, under100contigMycos_v12.

### Identification of phage-like regions

Accessory gene content present in *Mycobacterium* strains with 30 or fewer contigs was annotated with a database of HMM profiles consisting of archetypal phage genes as defined previously (30) and select pfams of integrases/transposases. The created database is also available at (https://osf.io/zt4n3/). A sliding window of 15 genes was applied over each genome, and regions containing one or more significant alignments to a profile in the database were recorded. Regions containing one or more phage genes were identified as phage-related regions and regions containing either an integrase or a transposase were identified as integrated elements. A BLASTn database of nucleotide sequences was constructed from representative phage and prophage genomes, and phage-like regions were labeled as likely defective prophages if there was significant alignment to one or more intact phage sequences. A similar strategy was used to identify phiRv1 and phiRv2 elements. Candidate PICIs were described simply as phage-like regions with both integration machinery and a major capsid protein gene, as the HMM profiles for these sequences are robust and likely to detect remote homologs; candidate PICIs were also discovered using SatelliteFinder (46). Homologs of major capsid proteins present in candidate PICIs were aligned with Clustal Omega (57) and alignments were trimmed with trimAI (58) , and a phylogeny was created with RAxML (59) using the PROTGAMMAGTR model.

### Phylogeny of *Mycobacterium* representatives

*Mycobacterium* assemblies listed as reference strains at PATRIC and GenBank were downloaded for all taxa listed in Table S3 with ≥3 members within the taxa. Assemblies were annotated with prodigal (60) and ortholog sequence clusters were generated using Mmseqs2 and PhaMMseqs (34) with 50% identity and 50% coverage. Amino acid sequences were aligned with Clustal omega (57) and trimmed with trimAI (58). Alignments for selected core sequence clusters were concatenated with a custom python script and RaxML was run on the concatenated alignment with the PROTGAMMAGTR model (59).

### Identification of attachment sites

Prophage core attachment sequences sites were identified and reported by DEPhT as described previously (30). For each genome analyzed, coordinates and core attachment site sequence data from reconstructions of *attB* by DEPhT were recorded for a relevant *Mycobacterium* reference strain. Consensus *attB* sites were identified by calculating the conserved coordinate positions for each set of overlapping *attB*s.

### Construction, transformation, and verification of integration vectors

Integration-proficient plasmid vectors were constructed from prophage sequences predicted by DEPhT to integrate at particular *attB* sites. The *attP* sites were reconstructed by circularizing the phage genome at the core attachment site sequences as reported by DEPhT. Synthetic DNA was ordered from Twist Bioscience containing the putative integrase together with the reconstructed *attP*, and inserted into plasmid vector pTwist Kan High Copy. *M. smegmatis* mc^2^155 competent cells were prepared as described previously (61). Competent cells of *M. abscessus* ATCC19977 were prepared by inoculating a culture 1:10 in 7H9 with 10% oleic albumin dextrose catalase (OADC) (vol/vol) and incubating at 37°C for 16–24 hours. Cultures were chilled on ice for 30 min, cells were pelleted by centrifugation at 5,000 × *g* for 10 min, washed in 10% glycerol (vol/vol), 250 mM sucrose, and 0.5 mM potassium acetate, and the volume lowered in a stepwise fashion as described previously. Plasmids were electroporated into *M. smegmatis* mc^2^155 and *M. abscessus* ATCC19977 as described previously and transformants recovered on selective media. Transformants were screened by PCR to determine if the plasmids integrated into the predicted *attB* sites.

### Identification and analysis of PESTs

To identify likely PEST systems, we searched for protein sequences related to polymorphic toxins described previously (29) and collected the sequence hits together with the surrounding genes encoding predicted WXG100 effector or immunity proteins. These sequence phamilies identified were used in an HMM-HMM profile search using HH suite (62) against all phams in identified prophages to find PEST cassettes components that are distinct but similar in sequence. To capture PEST systems with novel sequences, polymorphic toxin genes identified as belonging to PEST systems through sequence homology were used as the anchor in a gene neighborhood network analysis. A network was constructed using information about genes encoded adjacent to identified polymorphic toxin genes, where each node in the graph represents one nonredundant pham of genes. For each polymorphic toxin gene, those genes encoded upstream and downstream were identified in a breadth-first search (BFS) manner and added to the network, creating an edge between the pham of the polymorphic toxin gene and the adjacent gene. New nodes added to the graph were used as the additional anchors in further iterations of the search; all genes that are members of discovered phams were analyzed for upstream/downstream genes. This search encompassed all genes two ORFs upstream/downstream of polymorphic toxin genes, and nodes subject to BFS were continued until no new nodes were identified. Putative PEST systems found in the gene neighborhood network analysis were searched against databases at HHPred (56) for sequence similarity to known Type VII secreted proteins and other polymorphic toxin domains. All PEST systems were grouped into superfamilies by detecting shared domains through HMM-HMM profile alignments between phams of polymorphic toxins of PEST systems.

### Structural prediction of PESTs

Structures of PEST proteins were predicted with AlphaFold. For PEST1 and PEST2 superfamilies, structures were predicted of an artificial sequence created from a polymorphic toxin and its WXG100 family effector connected by a poly-glycine linker with 30 residues. Domains of structures of secretion signal domains were identified through amino acid conservation and shared domains of PEST superfamilies. Secretion signal domains of polymorphic toxins encoded by prophiGD43A-6 and prophiGD17-1 were compared to structures available at the PDB as well as AlphaFold predictions of proteins encoded by *Mycobacterium tuberculosis* using the DALI webserver (63). Structural predictions and alignments were visualized with PyMOL (available at https://pymol.org/2/).

### Mutagenesis of PEST cassettes

Non-toxic mutants of pLA7 were identified via random mutagenesis using Agilent *E. coli* XL1-Red and subsequent transformation of mutagenized plasmid into *M. smegmatis* mc^2^155. Competent cell of *E. coli* XL1-Red were prepared according to standard protocols provided with the product. Fifty nanograms of pLA2 and pLA7 was transformed into separate tubes of competent XL1-Red cells, incubated on ice for 30 min, and heat-shocked for 45 s in a 42°C water bath. Cells were recovered in 0.9 mL of pre-warmed SOC medium and incubated at 37°C, shaking for 1 hour. Nine hundred microliters of the mix was plated onto solid media with 20 µg/mL kanamycin and incubated for 24 hours. Colonies were scraped into a 10 mL volume of liquid medium with 20 µg/mL kanamycin and incubated at 37°C, shaking, for 48 hours. Mutagenized pLA2 and pLA7 plasmid DNA was extracted and 100 ng of wild type (WT) and mutagenized DNA was transformed into *M. smegmatis* mc^2^155 as described previously (61). PEST cassettes of mutagenized pLA7 transformants of *M. smegmatis* were PCR amplified and sequenced.

## Data Availability

Code for DEPhT and SPLICE used to identify prophages is available at github: http://github.com/chg60/DEPhT and http://github.com/laa89/SPLICE. *Mycobacterium* genomes are available at PATRIC, and the IDs are listed in Data set S1. Phamerator databases used for analysis, under100contigMycos_v5 and under100contigMycos_v12, are available at http://databases.hatfull.org/Published/.

## SUPPLEMENTAL MATERIAL

Data Set S1 msystems.00446-23-s0001.csv
*Mycobacterium* genome assemblies analyzed with DEPhT and SPLICE.

Data Set S2 msystems.00446-23-s0002.csv Prophages discovered in *Mycobacterium* genomes.

Data Set S3 msystems.00446-23-s0003.csv Likely defective prophages identified in *Mycobacterium* genomes.

Data Set S4 msystems.00446-23-s0004.csv Candidate PICI elements identified in *Mycobacterium* genomes.

Data Set S5 msystems.00446-23-s0005.csv phiRv-like elements identified in *Mycobacterium* genomes.

Supplemental Information msystems.00446-23-s0006.pdf Supplemental figures and tables, as well as captions to Data Sets S1 to S5.

ASM does not own the copyrights to Supplemental Material that may be linked to, or accessed through, an article. The authors have granted ASM a non-exclusive, world-wide license to publish the Supplemental Material files. Please contact the corresponding author directly for reuse.
