# Supplementary material for: The heterogenous and diverse population of prophages in Mycobacterium genomes
Source: mSystems. 2023 Oct 4;8(5):e00446-23. doi: 10.1128/msystems.00446-23 (PMC10654092; doi:10.1128/msystems.00446-23)
Supplement: Supplemental Information — Supplemental figures and tables, as well as captions to Data Sets S1 to S5. [file msystems.00446-23-s0006.pdf]

Supplementary Information

Table S1. Prophage abundance in NTM species that carry prophages.

Table S2. *attB* sites in *M. abscessus.*

Table S3. Integration-Proficient plasmids used in this study.

Table S4. Primers used in this study.

Supplementary Dataset 1. *Mycobacterium* genome assemblies analyzed with DEPhT and SPLICE. A complete listing of all *Mycobacterium* genomes from PATRIC and analyzed for prophage content. Genomes of *Mycobacteriaceae* were downloaded from PATRIC if the assembly was in 100 or fewer contigs. For each genome, the PATRIC genome ID, taxon ID, number of contigs, genome assembly status, genome genus, and genome species are listed. Additionally, the number of prophages identified, and their assigned clade as reported in this study, is listed.

Supplementary Dataset 2. Prophages discovered in *Mycobacterium* genomes. A table listing whole prophages identified after analyzing genomes of *Mycobacterium* with DEPhT. Contigs of sequence assemblies downloaded from PATRIC (Supplementary Dataset 1), were assigned individual labels as *PATRIC_ID_Contig#*. Rows describe the strain and contig for each prophage discovered, along with the length of that contig, the start, end, and strand (1, -1) of the identified prophage, the ID assigned to the prophage by DEPhT, and the name reassigned to the prophage based on its host strain. Additional columns highlight the cluster assigned to the prophage by PhamClust, whether or not the prophage could be identified as a duplicate of another prophage, and what the ID of that identical prophage is, where applicable. Prophages were omitted from the database if they showed evidence of improper extraction or truncation by the ends of contigs. In these cases, Prophage Cluster was marked as OMITTED and excluded from clustering and gene content analyses.

Supplementary Dataset 3. Likely defective prophages identified in *Mycobacterium* genomes. A table listing likely defective prophages in *Mycobacterium* genomes with 30 contigs or fewer. Likely defective prophages were identified by searching for nucleotide and protein product sequence similarity to intact phage genomes in the accessory gene content of *Mycobacterium.* Data rows describe the assigned region ID, host strain, contig ID, start, and end of the identified region. A count of homologs to archetypal phage proteins is listed under Num_Products, and their product descriptions are listed under Products, delimited by semicolons. PEST_PT_homologs lists if any homologs to PEST system polymorphic toxins were identified in the defective phage genome, and if so, the phamily of the homologous PEST system polymorphic toxins.

Supplementary Dataset 4. Candidate PICI elements identified in *Mycobacterium* genomes. A table listing candidate PICIs in *Mycobacterium* genomes with 30 contigs or fewer. Candidate PICIs were identified by searching for homologs to integrase/transposase genes as well as phage major capsid proteins in the accessory gene content of *Mycobacteria*. Data rows describe the assigned region ID, host strain, contig ID, start, and end of the identified region. A count of homologs to archetypal phage proteins is listed under Num_Products, and their product descriptions are listed under Products, delimited by semicolons. PEST_PT_homologs lists if any homologs to PEST system polymorphic toxins were identified in the defective phage genome, and if so, the phamily of the homologous PEST system polymorphic toxins. SatelliteFinder was used to analyze all the identified regions, and the SatelliteFinder Type is reported where applicable, indicating the amount of PICI homologs identified by SatelliteFinder.

Supplementary Dataset 5. phiRv-like elements identified in *Mycobacterium* genomes. A table listing phiRv-like elements in *Mycobacterium* genomes with 30 contigs or fewer. phiRv-like elements were identified by searching for homologs to integrase/transposase genes as well as nucleotide similarity to phiRv1 and phiRv2 from the genome of *Mycobacterium tuberculosis* H37Rv. Data rows describe the assigned region ID, host strain, contig ID, start, and end of the identified region. A count of homologs to archetypal phage proteins is listed under Num_Products, and their product descriptions are listed under Products, delimited by semicolons.

Supplementary Figure Legends

Figure S1. Identification of new PEST systems using both homology searches and iterative searches for gene neighborhoods. A. Schematic representation of the gene neighborhood analysis used to identify Phage-encoded ESX Secreted Toxin (PEST) cassettes. In brief, all sequence-related groups of genes closely linked to known PESTs were identified, and because PEST systems can have different sets of WXG100 effectors, polymorphic toxins, and immunity proteins, many sequence groups can be associated with one polymorphic toxin. These genes were used in a gene neighborhood analysis where genes were considered associated with each other (or in the same neighborhood as) if two genes were encoded adjacent to each other. A complete search of genes associated with described polymorphic toxins was then performed. The sequence homologs of these genes were then cataloged, and the process iterated, finding all genes associated with this collection. The associations between all genes discovered, as well as the homologs of these genes, were displayed in a directed graph and novel PEST systems were identified through traversing this graph. New PEST systems discovered, displayed in the figure as ORFs with varying color, were used to anchor further searches and this process iterated until no new sequence clusters were found. **B**. A

gene neighborhood network of all PEST genes discovered as illustrated in Figure 5, Figure S1A, and described in the figure legend. **C**. PEST sequence cluster HMM profiles were aligned against each other. HMM-HMM profile alignments with greater than 90% probability and up to 100% are displayed, where edges between nodes are colored on a gradient between orange and red based on their probability. PEST sequence clusters connected to each other by HMM-HMM profile alignments of their secretion domain were grouped into PEST superfamilies. **D**. A cartoon of select PEST HMM-HMM profile alignments for PEST1, PEST2, and PEST3 phams. PEST superfamily sequence similarity is localized to the n-terminal secretion signal domain of polymorphic toxin proteins. Secretion signal domain homology between polymorphic toxin phams is indicated by lines between WXG domains (blue). Numbers adjacent to domains indicate the start and end columns for the respective HMM profile.

Figure S2. Examples of integrated elements, phage-related regions, and defective prophages. A. A venn diagram illustrating of the logical relationship between identified regions. Integrated elements encode one or more integrase/transposase genes while phage-related regions encode at least one archetypal phage gene. Phage-related regions often encode integrase genes, although regions exist containing phage-like genes without an adjacent integrase/transposase. Likely defective prophages and candidate PICIs are subgroups of phage-related regions; candidate PICIs contain both integrase/transposase genes and major capsid genes while likely defective prophages encode a phage-like gene and share nucleotide sequence similarity with an intact phage genome. **B.** A phylogenetic tree of the major capsid proteins of candidate PICIs together with capsid proteins of *Mycobacterium* phages and prophages. Phage and prophage sequences are colored white on the outer ring, and the colors in the outer ring represent Mycobacterium clades containing the PICIs. The cluster designations of phages and prophages are annotated next to relevant tree branches on the phylogeny. **C.** Genome maps of an integrated element and a likely defective prophage. The

integrated element within GD68A contains no archetypal phage genes (other than integrases), while the displayed region in GD91 encodes phage structural and DNA packaging genes. The region encoded by GD91 shares significant nucleotide sequence similarity with intact cluster M *M. abscessus* prophages, such as prophiT49-2 but appears to have lost ~52 kbp downstream of the portal gene.

Table S1. Prophage abundance in NTM species that carry prophages

Species[1]	# genomes[2]	# genomes ≥1 prophage[3]	# prophages[4]	Mean #prophages[5]	# Unique Prophages[6]
Mycobacteroides_sp._H063	1	1	2	2	2
Mycobacteroides_sp._H001	1	1	2	2	2
Mycobacteroides_sp._H054	1	1	2	2	2
Mycobacteroides_sp._HXVII	1	1	2	2	2
Mycobacteroides_sp._HXXIII	1	1	2	2	2
Mycobacteroides_sp._H079	1	1	2	2	2
Mycobacteroides_sp._H002	1	1	2	2	2
Mycobacterium_sp._D16Q16	1	1	2	2	1
Mycobacteroides_sp._H101	1	1	2	2	2
Mycobacterium_sp._MAC_080597_8934	1	1	1	1	1
Mycobacterium_sp._D16R24	1	1	1	1	1
Mycolicibacterium_sp._CBMA_293	1	1	1	1	1
uncultured_Mycobacterium_sp.	1	1	1	1	1
Mycobacterium_sp._365mftsu	1	1	1	1	1
Mycobacterium_sp._IS-836	1	1	1	1	1
Mycobacterium_sp._D17A2	1	1	1	1	0
Mycobacterium_sp._D16Q14	1	1	1	1	1
Mycobacterium_sp._Root265	1	1	1	1	1
Mycolicibacterium_sp._CBMA_335	1	1	1	1	0
Mycolicibacterium_sp._CBMA_360	1	1	1	1	1
Mycobacterium_sp._MHSD3	1	1	1	1	1
Mycobacteroides_sp._H003	1	1	1	1	1
Mycobacterium_sp._BK086	1	1	1	1	1
Mycolicibacterium_sp._CBMA_213	1	1	1	1	0
Mycobacteroides_sp._H092	1	1	1	1	1
Mycobacteroides_sp._H072	1	1	1	1	1
Mycolicibacterium_sp._BK634	1	1	1	1	1
Mycobacterium_sp._UNCCL9	1	1	1	1	1
Mycobacterium_sp._D16Q20	1	1	1	1	1
Mycolicibacterium_sp._CBMA_311	1	1	1	1	1
Mycobacterium_sp._AZCC_0083	1	1	1	1	1
Mycobacterium_sp._DL440	1	1	1	1	1
Mycolicibacterium_sp._CBMA_230	1	1	1	1	0
Mycobacterium_sp._PYR10	1	1	1	1	1
Mycobacterium sp. SWH-M5	1	1	1	1	0
Mycolicibacterium sp. CBMA 361	1	1	1	1	0
Mycobacteroides sp. H110	1	1	1	1	0
Mycobacterium sp.	1	1	1	1	0
Mycobacterium_sp._NRRL_B-3805	2	1	1	0.5	1
Mycobacterium_sp._djl-10	4	1	1	0.25	1
TOTAL[7]	44	40	49	1.114	40

[1]Unspeciated strains in the genus *Mycobacterium* for which at least one genome entry contains at least one prophage.
[2]The total numbers of genomes from one group of strains analyzed with DEPhT and SPLICE.
[3]The total numbers of genomes from one group of strains with one or more prophages identified using DEPhT and SPLICE.
[4]The total numbers of intact prophages identified using DEPhT and SPLICE for each group of strains.
[5]The average numbers of prophages for each species are shown.
[6]The total numbers of prophages with unique sequences identified using DEPhT.
[7]The total numbers of genomes, prophages, and unique prophages in strains not speciated as labelled at PATRIC.

Table S2. *attB* sites in *M. abscessus*

attB	ATCC19977 coordinates	Core sequence[1]	Int	Prophage Cluster(s)	attB Location
attB-1	233517..233550	CAGAAGGTTAGGGGTTCGAATCCCTTCGGGC GCA	Int-Y	HK, HN	Mab_t5006; tRNA-Arg(ACG)
attB-24	250045..250078	CAGAAGGTTAGGGGTTCGAATCCCTTCGGGC GCA	Int-Y	HK, HN	Intergenic Mab_0245c-Mab_0246
attB-2	490929..490956	TGAATAGGTCAGGGGTTCGATTCCCCTG	Int-Y	HB, HG	Mab_t5010; tRNA-Thr(CGT)
attB-23	770382..770396	GCGGATTAAAAGTCC	Int-Y	HR, L	Mab_0771c; predicted major transport protein
attB-25	779048..779063	TGCGGATTAAAAGTCC	Int-Y	L	Mab_t5012c; tRNA-Lys(TTT)
attB-3	1102725..1102749	AGGGGTTCGATTCCCCTTAGCTCCA	Int-Y	HD	Mab_t5017; tRNA-Ala(CGC)
attB-15	1191302..1191309	CTTAAAAT	Int-Y	HD	Mab_t5019; tRNA-Leu(TAA)
attB-19	1535943..1535953	AGTACAACGTG	Int-S	singleton	Mab_1515c ; alkyl hydroperoxide reductase
attB-18	1550160..1550203	GTGCGCCGTCAGGGGCTCGAACCCCGGACC CGCTGATTAAGAGT	Int-Y	HR	Mab_t5022c ; tRNA-Lys(CTT)
attB-26	1582496..1582539	CAGCGCCCCCGGCAGGAATCGAACCTGCGAC CTAGGGATTAGAA	Int-Y	HA	Mab_t5024c; tRNA-Arg(TCT)
attB-4	1739385..1739418	CGGACGGTTATTGGTTCGAGTCCAATCGGGG GAG	Int-Y	HE	Mab_t5027; tRNA-Asn(GTT)
attB-5	1754373..1754430	GGGGCGGTAGCTCAGTTGGTTAGAGCCGTGG ACTCATAATCC	Int-Y	HA	Mab_t5028; tRNA-Met(CAT)
attB-21	1849335..	AC	Int-S	A	Mab_1851;probable acyl-CoA dehydrogenase FadE
attB-20	1885773..1885795	GGGTTCGAAACCCTCCGCGCCCA	Int-Y	HH	Mab_t5029; tRNA-Val(TAC)
attB-22	2089032..2089079	GTGCGCCGTCAGGGTTTCGAACCCCAGACCC GCTGATTAAGAGTCAGC	Int-Y	HR	Mab_t5030; tRNA-Lys(CTT)
attB-6	2431270..2431328	GGTAGCGCACTTGACTGGGGGTCAAGTGGTC GCAGGTTCAAATCCTGTCAGCCCGACCA	Int-Y	HP	tRNA-Pro(GGG)
attB-7	2502031..2502035	TACGA	Int-S	A	Mab_2445; AraC transcriptional regulator
attB-8	3039280..3039294	CAACTCAATTAGTCT	Int-Y	HB	Mab_2979; peptide methionine sulfoxide reductase-related protein
attB-9	3265144..3265156	TAGTCGATATAGG	Int-S	M	Mab_3230c
attB-17	3302842..3302850	AGCCCCTTG	Int-S	M	Mab_3265c
attB-10	3491832..3491850	TATGGTGGAGCTAAGGGGA	Int-Y	HC	Mab_t5041c; tRNA-Ala(GGC)
attB-11	3513406..3513428	GTGGAGCTGCCGGGAATTGAACC	Int-Y	HC	tmRNA
attB-28	3527276..3527285	CTTACCGCTT	Int-Y	HB	Mab_t3485; monophosphatase
attB-16	3537980..3538036	CTCCGGCCTACGTACCTAGTAGCGGGGACAG GATTTGAACCTGCGACCTCTGGGTTA	Int-Y	HE	Mab_t5042c; tRNA-Met(CAT)
attB-29	3755483..3755487	TTCC	Int-S	A	Mab_3702; TrmH tRNA methyltransferase
attB-12	3869544..3869551	TACTTTCG	Int-Y	HH	Mab_3823
attB-27	3965457..3965500	GACGGATTTACAGTCCGCTCCCATTGGCCGC TCGGGCAACCCGC	Int-Y	HT	Mab_t5046c;tRNA-Tyr(GTA)
attB-13	3995694..3995713	TTCTTACTCGTGAGTAAGAA	Int-Y	HC	Mab_3947; oxidoreductase
attB-14	4519109..4519126	ATTCTTACTCTGGAGTAA	Int-Y	HO/HH	Intergenic Mab_4442c-Mab4443

[1]The common core sequence shared between *attP*, *attB*, *attL* and *attR* for all discovered prophages is shown; mismatches are in bold type.

Table S3. Integration-Proficient plasmids used in this study

Plasmid	attB	Int	Reference	Description
pMH94	attB-1, *M. smegmatis* mc^2155	Int-Y	Lee, MH., et. al 1993	Kan-marked integration-proficient vector derived from the *attP/int* of Mycobacteriophage L5
pIF03	attB-26; *M. abscessus* ATCC19977	Int-Y	this study	Kan-marked integration-proficient vector derived from the *attP/int* of prophi1962118.185_4-1
pIF04	attB-28; *M. abscessus* ATCC19977	Int-Y	this study	Kan-marked integration-proficient vector derived from the *attP/int* of prophi319705.109_14-4
pIF05	attB-29; *M. abscessus* ATCC19977	Int-S	this study	Kan-marked integration-proficient vector derived from the *attP/int* of prophi1185650.948_10-1
pLA2	attB-1, *M. smegmatis* mc^2155	Int-Y	this study	pMH94-based vector which contains the PEST cassette from prophiGD43A-6
pLA3	attB-1, *M. smegmatis* mc^2155	Int-Y	this study	pMH94-based vector which contains the PEST cassette from prophiGD05-3
pLA5	attB-1, *M. smegmatis* mc^2155	Int-Y	this study	pMH94-based vector which contains the PEST cassette from prophiGD08-3
pLA6	attB-1, *M. smegmatis* mc^2155	Int-Y	this study	pMH94-based vector which contains the PEST cassette from prophiGD17-1
pLA9	attB-1, *M. smegmatis* mc^2155	Int-Y	this study	pMH94-based vector which contains the PEST cassette from prophiGD03-1
pLA7	attB-1, *M. smegmatis* mc^2155	Int-Y	this study	pMH94-based vector which contains the PEST cassette from prophiGD43A-6 without its cognate immunity protein
pLA14	attB-1, *M. smegmatis* mc^2155	Int-Y	this study	pMH94-based vector which contains the PEST cassette from prophiGD05-3 without its cognate immunity protein
pLA15	attB-1, *M. smegmatis* mc^2155	Int-Y	this study	pMH94-based vector which contains the PEST cassette from prophiGD17-1 without its cognate immunity protein
pLA16	attB-1, *M. smegmatis* mc^2155	Int-Y	this study	pMH94-based vector which contains the PEST cassette from prophiGD08-3 without its cognate immunity protein
pLA17	attB-1, *M. smegmatis* mc^2155	Int-Y	this study	pMH94-based vector which contains the PEST cassette from prophiGD03-1 without its cognate immunity protein
pLA27	attB-1, *M. smegmatis* mc^2155	Int-Y	this study	pLA2-based vector where the encoded polymorphic toxin cassette has an engineered mutation where residues 2 to 456 have been deleted.
pLA28	attB-1, *M. smegmatis* mc^2155	Int-Y	this study	pLA2-based vector where the encoded polymorphic toxin cassette has an engineered mutation where residues 2 to 474 have been deleted.
pLA29	attB-1, *M. smegmatis* mc^2155	Int-Y	this study	pLA2-based vector where the encoded polymorphic toxin cassette has an engineered mutation where residues 2 to 511 have been deleted.
pLA31	attB-1, *M. smegmatis* mc^2155	Int-Y	this study	pLA7-based vector where the encoded polymorphic toxini cassette has an engineered mutation where residues 2 to 456 have been deleted.
pLA32	attB-1, *M. smegmatis* mc^2155	Int-Y	this study	pLA7-based vector where the encoded polymorphic toxin cassette has an engineered mutation where residues 2 to 474 have been deleted.
pLA33	attB-1, *M. smegmatis* mc^2155	Int-Y	this study	pLA7-based vector where the encoded polymorphic toxin cassette has an engineered mutation where residues 2 to 511 have been deleted.
pLA34	attB-1, *M. smegmatis* mc^2155	Int-Y	this study	pLA2-based vector where the encoded polymorphic toxin has an engineered mutation where residues 2 to 433 have been deleted.
pLA35	attB-1, *M. smegmatis* mc^2155	Int-Y	this study	pLA7-based vector where the encoded polymorphic toxin has an engineered mutation where residues 2 to 433 have been deleted.

Table S4. Primers used in this study

Primer	Sequence	Description
M13	GTAAAACGACGGCCAGT	Common sequencing primer, used for amplification across the junction of *attL/attR* for integrated plasmids pIF03, pIF04, and pIF05
pIF03_attR_R	AAAGCCGCCAGCAGAAA	Reverse primer which anneals to a nucleotide sequence in *M. abscessus* ATCC19977 downstream of *attR* for integrated pIF03
pIF04_attR_R	GGGGTAGGTCTTGTTCTTGT	Reverse primer which anneals to a nucleotide sequence in *M. abscessus* ATCC19977 downstream of *attR* for integrated pIF04
pIF05_attL_F	AACACCGAATCAAGCAAG	Forward primer which anneals to a nucleotide sequence in *M. abscessus* ATCC19977 upstream of *attL* for integrated pIF05
MAB_eccE4_52_F	ATCCAGATCATGGTGA	Forward primer which anneals to an *M. abscessus*-specific gene, EccE4
MAB_eccE4_470_R	TGATGACGTCGATGCT	Reverse primer which anneals to an *M. abscessus*-specific gene, EccE4
pTwistKH_191_F	AGCAAAAGGCCAGCAAAA	Forward primer which anneals to the backbone of plasmids pIF03, pIF04, and pIF05.
pTwistKH_847_R	CAGACCCCGTAGAAAAGA	Reverse primer which anneals to the backbone of plasmids pIF03, pIF04, and pIF05.
prophiGD43A-6_PT_F	TTGTAAAACGACGGCCAGTGAATTCACACCCG AACCACTAAGTG	Forward primer used to amplify and clone the PEST cassette of prophiGD43A-6
prophiGD43A-6_PT_R	ATCCCCGGGTACCGAGCTCGAATTCCTACTGC CCCTTGATGGC	Reverse primer used to amplify and clone the PEST cassette of prophiGD43A-6
prophiGD05-3_PT_F	TTGTAAAACGACGGCCAGTGAATTCGCCCCGA CTCCGACTTGC	Forward primer used to amplify and clone the PEST cassette of prophiGD05-3
prophiGD05-3_PT_R	ATCCCCGGGTACCGAGCTCGAATTCCTACAGC TCCTCACCGATGTCG	Reverse primer used to amplify and clone the PEST cassette of prophiGD05-3
prophiGD08-3_PT_F	TTGTAAAACGACGGCCAGTGAATTCCGCCCCC ATGCCTACAAC	Forward primer used to amplify and clone the PEST cassette of prophiGD08-3
prophiGD08-3_PT_R	ATCCCCGGGTACCGAGCTCGAATTCCTAGCGC GTCTGGCGTGC	Reverse primer used to amplify and clone the PEST cassette of prophiGD08-3
prophiGD17-1_PT_F	TTGTAAAACGACGGCCAGTGAATTCGAGAAGG TCAGGGGTTCG	Forward primer used to amplify and clone the PEST cassette of prophiGD17-1
prophiGD17-1_PT_R	ATCCCCGGGTACCGAGCTCGAATTCCTATGCC TTCCAGTAGTGG	Reverse primer used to amplify and clone the PEST cassette of prophiGD17-1
prophiGD03-1_PT_F	TTGTAAAACGACGGCCAGTGAATTCGACAGGT TCCCGTGTACC	Forward primer used to amplify and clone the PEST cassette of prophiGD03-1
prophiGD03-1_PT_R	ATCCCCGGGTACCGAGCTCGAATTCTCACATA GCGCGCGGATAC	Reverse primer used to amplify and clone the PEST cassette of prophiGD03-1
pLA2_Imm-_SDM_F	GAATTCGAGCTCGGTACC	Forward primer used in site-directed mutagenesis to delete the immunity protein gene encoded by pLA2
pLA2_Imm-_SDM_R	CGATCTTCCTAAATGGTCG	Reverse primer used in site-directed mutagenesis to delete the immunity protein gene encoded by pLA2
pLA3_Imm-_SDM_F	GTGGTTGACATCACTCGGTTC	Forward primer used in site-directed mutagenesis to delete the immunity protein gene encoded by pLA3
pLA3_Imm-_SDM_R	TCATCCAAGGGGCGGTCC	Reverse primer used in site-directed mutagenesis to delete the immunity protein gene encoded by pLA3
pLA5_Imm-_SDM_F	GTCCGAATACCGGCGATT	Forward primer used in site-directed mutagenesis to delete the immunity protein gene encoded by pLA5
pLA5_Imm-_SDM_R	TCATGGTTTCGGAGTGTAG	Reverse primer used in site-directed mutagenesis to delete the immunity protein gene encoded by pLA5
pLA6_Imm-_SDM_F	TGCATCGCCAAGGGGCCG	Forward primer used in site-directed mutagenesis to delete the immunity protein gene encoded by pLA6

Primer	Sequence	Description
pLA6_Imm-_SDM_R	TCATTCCGCAGGAACCTCCACAAC	Reverse primer used in site-directed mutagenesis to delete the immunity protein gene encoded by pLA6
pLA9_Imm-_SDM_F	GAATTCGAGCTCGGTACC	Forward primer used in site-directed mutagenesis to delete the immunity protein gene encoded by pLA9
pLA9_Imm-_SDM_R	TTCTTCCCCTCCTAATTG	Reverse primer used in site-directed mutagenesis to delete the immunity protein gene encoded by pLA9
pLA2_PT_2_456del_F	GACCATCCCGCACCTGCG	Forward primer used in site-directed mutagenesis to delete residues 2 to 456 of the polymorphic toxin gene encoded by pLA2
pLA2_PT_2_456del_R	CATGAACTCATCGAGCGTCGTC	Reverse primer used in site-directed mutagenesis to delete residues 2 to 456 of the polymorphic toxin gene encoded by pLA2
pLA2_PT_2_474del_F	AGCGACAATCTGCCGCAT	Forward primer used in site-directed mutagenesis to delete residues 2 to 474 of the polymorphic toxin gene encoded by pLA2
pLA2_PT_2_474del_R	CATGAACTCATCGAGCGTC	Reverse primer used in site-directed mutagenesis to delete residues 2 to 474 of the polymorphic toxin gene encoded by pLA2
pLA2_PT_2_511del_F	TTCAGCCAGACACCCCCA	Forward primer used in site-directed mutagenesis to delete residues 2 to 511 of the polymorphic toxin gene encoded by pLA2
pLA2_PT_2_511del_R	CATGAACTCATCGAGCGTCG	Reverse primer used in site-directed mutagenesis to delete residues 2 to 511 of the polymorphic toxin gene encoded by pLA2
pLA2_PT_2_433del_F	GACCTCACCGGCACCGAA	Forward primer used in site-directed mutagenesis to delete residues 2 to 433 of the polymorphic toxin gene encoded by pLA2
pLA2_PT_2_433del_R	CATGAACTCATCGAGCGTCG	Reverse primer used in site-directed mutagenesis to delete residues 2 to 433 of the polymorphic toxin gene encoded by pLA2
pLA2_2023_F	CAAGACCAAACCAGCCAA	For sequencing the 3' end and downstream of the polymorphic toxin gene encoded by pLA2
pLA2_2863_R	CGAGCGTATCCAAAAACAA	For sequencing the 5' end and upstream of the polymorphic toxin gene encoded by pLA2
pLA2_3525_F	GTTTGGTGAGTGGATGAT	For sequencing the 3' end and downstream of the immunity protein gene encoded by pLA2
pLA2_3658_R	TTGTTGCGTGAATGTCAG	For sequencing the 5' end and upstream of the immunity protein gene encoded by pLA2

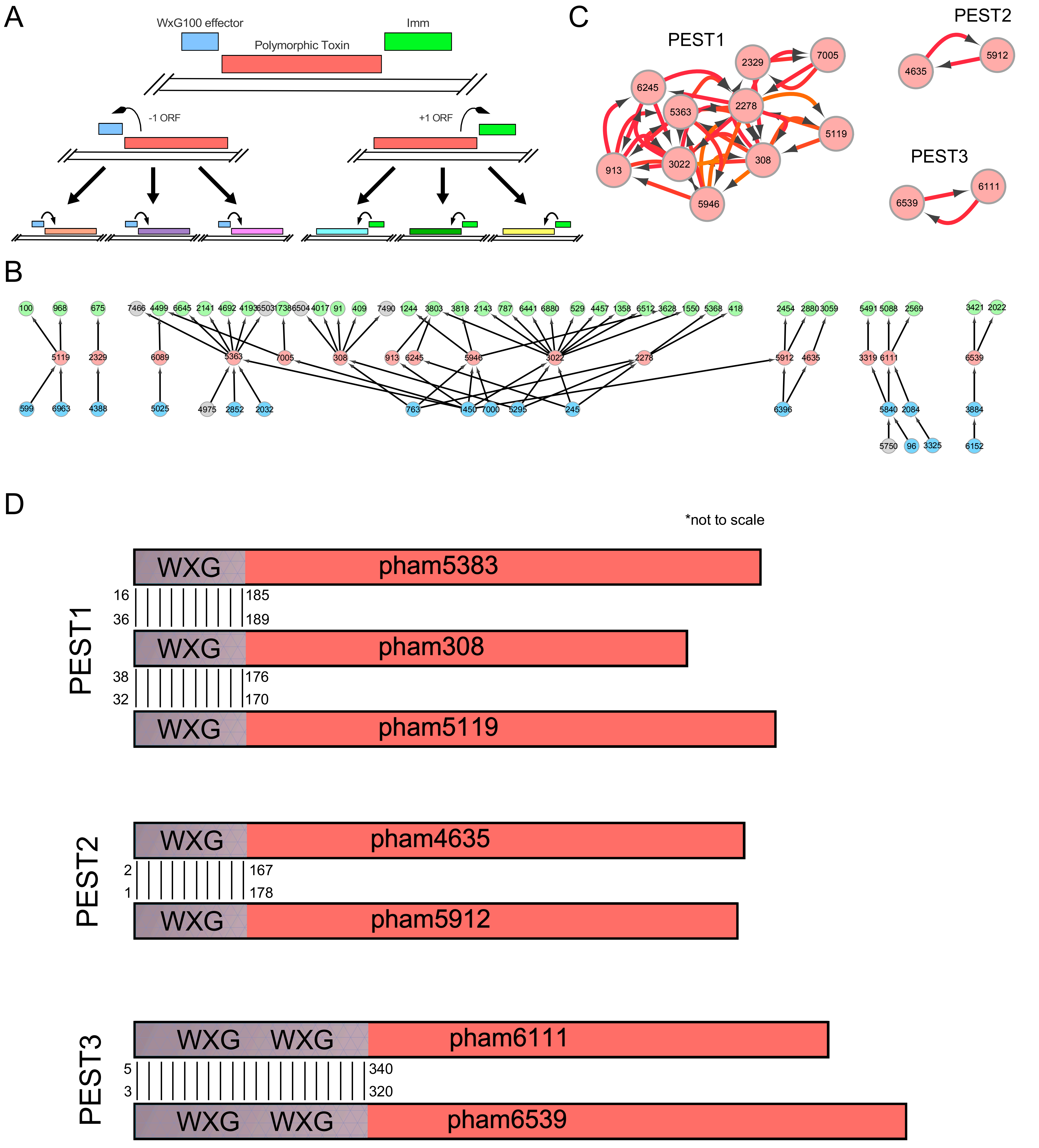
A
WxG100 effector
Imm
Polymorphic Toxin
-1 ORF
+1 ORF
B
C
PEST1
PEST2
PEST3
D
*not to scale
WXG
pham5383
pham308
pham5119
pham4635
pham5912
pham6111
pham6539

Figure S1

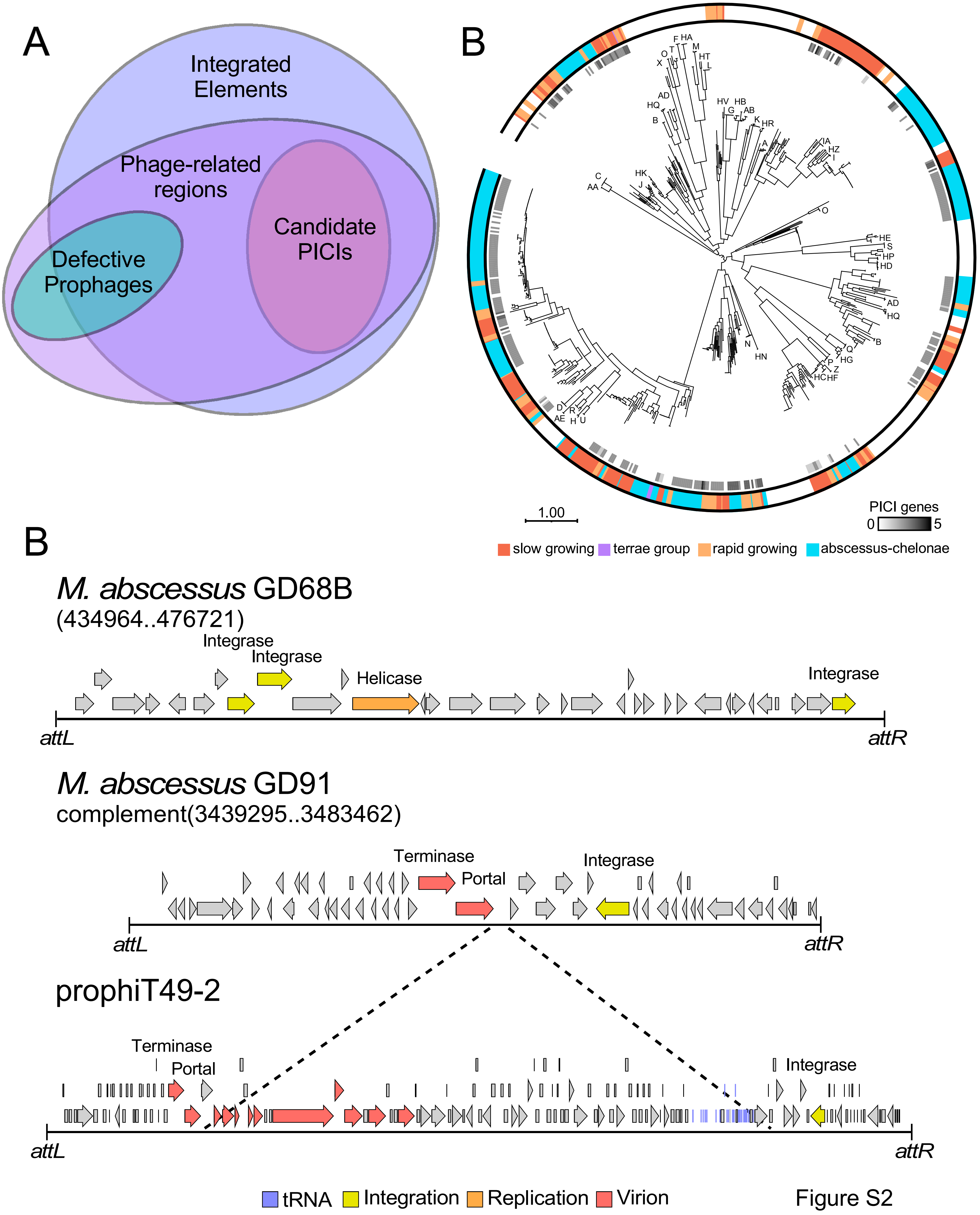
A
Integrated Elements
Phage-related regions
Candidate PICIs
Defective Prophages
B
1.00
PICI genes
0
5
slow growing
terrae group
rapid growing
abscessus-chelonae
B
M. abscessus GD68B
(434964..476721)
Integrase
Integrase
Helicase
Integrase
attL
attR
M. abscessus GD91
complement(3439295..3483462)
Terminase
Portal
Integrase
attL
attR
prophiT49-2
Terminase
Portal
Integrase
attL
attR
tRNA
Integration
Replication
Virion

Figure S2
